# Supplementary material for: Environment modulates protein heterogeneity through transcriptional and translational stop codon readthrough
Source: Nat Commun. 2024 May 24;15:4446. doi: 10.1038/s41467-024-48387-x (PMC11126739; doi:10.1038/s41467-024-48387-x)
Supplement: Supplementary file 1 — Supplementary Information [file 41467_2024_48387_MOESM1_ESM.pdf]

Supplementary Information

ENVIRONMENT MODULATES PROTEIN HETEROGENEITY THROUGH TRANSCRIPTIONAL AND TRANSLATIONAL STOP CODON READTHROUGH

Maria Luisa Romero Romero[1,2,*], Jonas Poehls[1,2], Anastasiia Kirilenko[1,2], Doris Richter[1,2], Tobias Jumel[1], Anna Shevchenko[1], Agnes Toth-Petroczy[1,2,3,*]

[1]Max Planck Institute of Molecular Cell Biology and Genetics, 01307 Dresden, Germany
[2]Center for Systems Biology Dresden, 01307 Dresden, Germany
[3]Cluster of Excellence Physics of Life, TU Dresden, 01062 Dresden, Germany

*Corresponding authors: romeroro@mpi-cbg.de and toth-petroczy@mpi-cbg.de

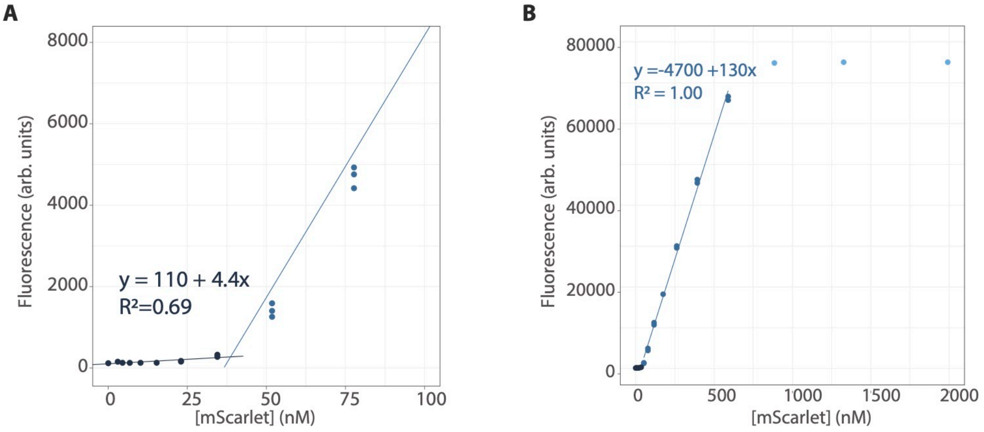

Supplementary Figure 1. Fluorescence measurements within the linear dynamic range of the microscope (270-7000 arb. units). A) The calibration curve range of mScarlet from 0-100 mM. The minimum concentration of mScarlet that we could determine with fluorescence measurements was 38 nM. **B)** A full range of the mScarlet calibration curve, from 0 -2000 mM. Saturation of the fluorescence signal above 70000 arb. units define the upper limit of the dynamic range. Source data is provided as the Source data file.

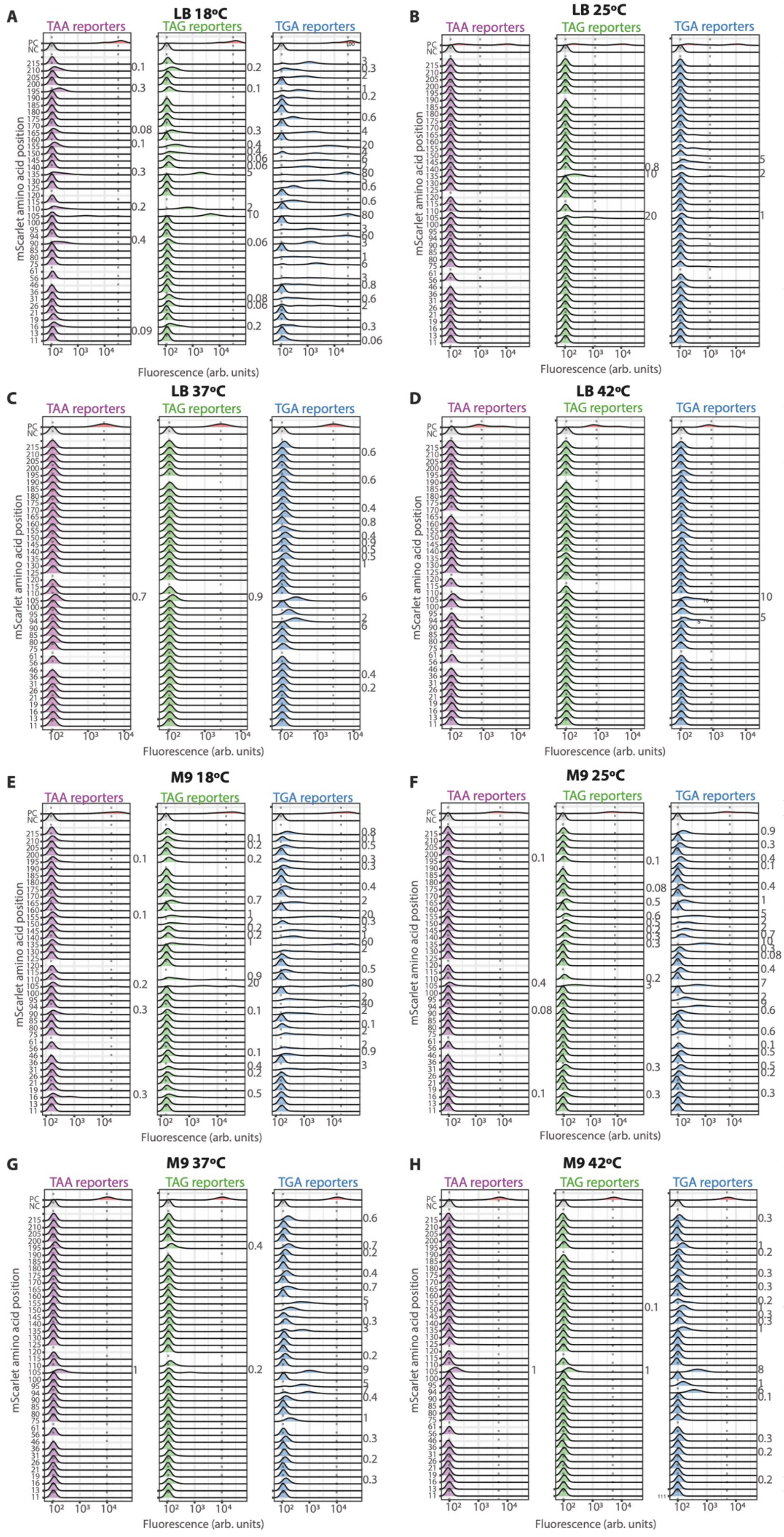

Supplementary Figure 2. Visualization and quantification of stop codon readthrough events in *E.coli* using fluorescence reporters. Fluorescence intensity distributions displayed by the *E.coli* cells, transformed with each library's reporters and grown at: **A)** 18℃ in rich media, **B)** 25℃ in rich media, **C)** 37℃ in rich media, **D)** 42℃ in rich media, **E)** 18℃ in minimum media, **F)** 25℃ in minimum media, **G)** 37℃ in minimum media, and **H)** 42℃ in minimum media. The propensity of SCR, calculated as the percentage of the median fluorescence compared with the positive control, is shown for the distributions with a median fluorescence higher than the negative control. Non-optimal growth temperatures and nutrient scarcity increase protein synthesis inaccuracy. Each distribution is derived from one replicate and 21 to 37029 cells. Rich media: LB and minimum media: M9 media supplemented with 0.4% glycerol, 0.2% casaamino acids, 1mM thiamine hydrochloride, 2 mM $MgSO_4$, and 0.1 mM $CaCl_2$. Source data is provided as Source data file.

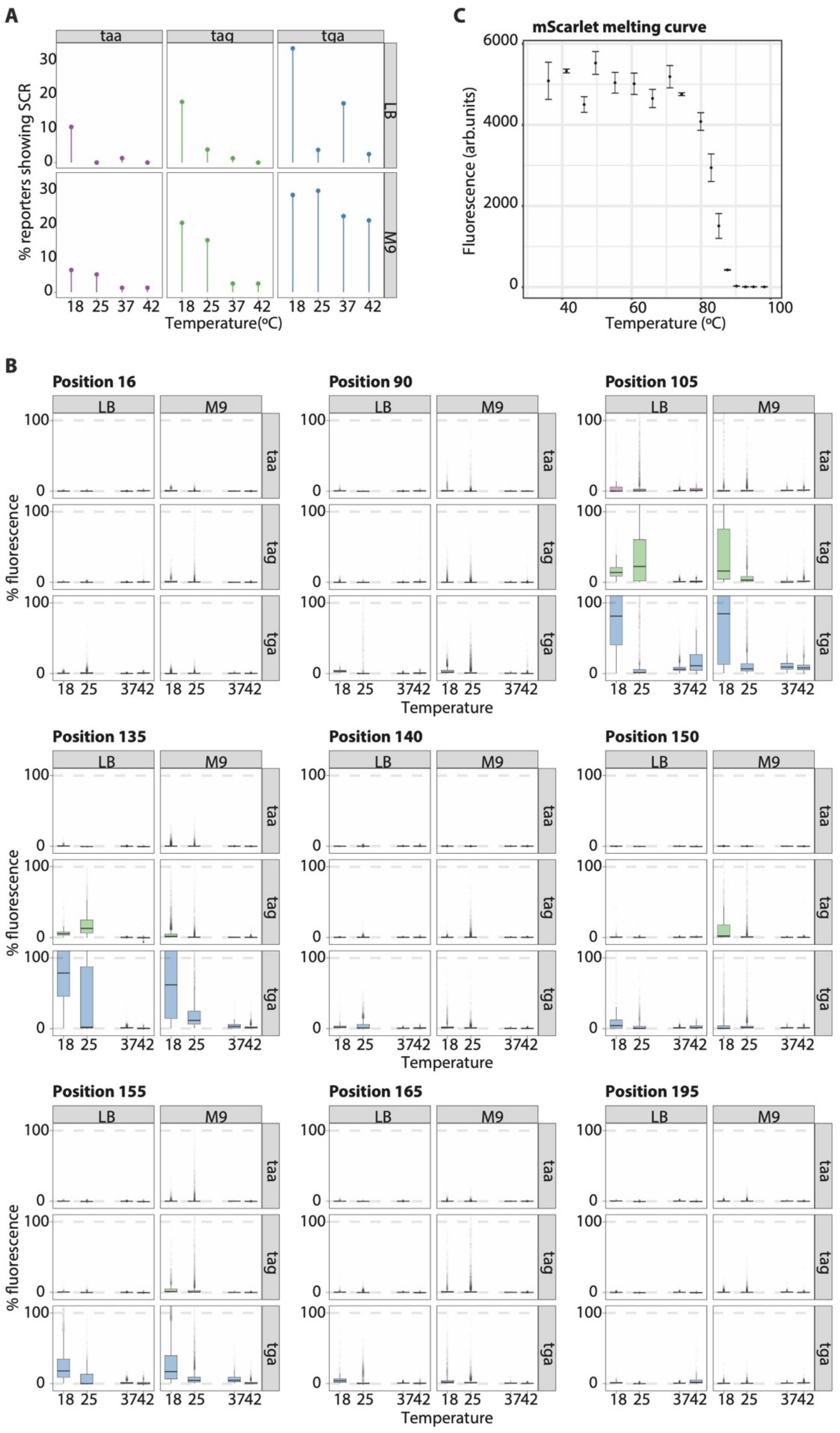

Supplementary Figure 3. Non-optimal growth temperatures and nutrient scarcity promote stop codon readthrough (SCR). A) More SCR events occur at lower temperatures, with TGA reporters displaying a higher incidence compared to TAG and TAA reporters. We considered those reporters

with a median fluorescence higher than the threshold defined as the median fluorescence plus two standard deviations of the NC. **B)** Box plots summarising fluorescence distributions of *E. coli* cells expressing nine selected reporters grown under various conditions, highlighting: i) increased errors in minimum media (M9) compared to rich media (LB), ii) TAA as the most accurate stop codon and TGA as the least accurate, and iii) higher SCR events at low temperatures and in minimum media. Y-axis represents median fluorescence relative to the positive control (PC). The box extends from the 25th to 75th percentile of the data and the horizontal line inside the box represents the 50th percentile of the data. The whiskers extend from the minimum to the maximum value within 1.5 times the interquartile range (IQR) from the lower and upper quartiles, respectively. **C) mScarlet thermostability assay.** mScarlet remained functional, i.e., fluorescent, until 70°C (mean and standard deviation of three replicates are shown). Source data is provided as Source data file.

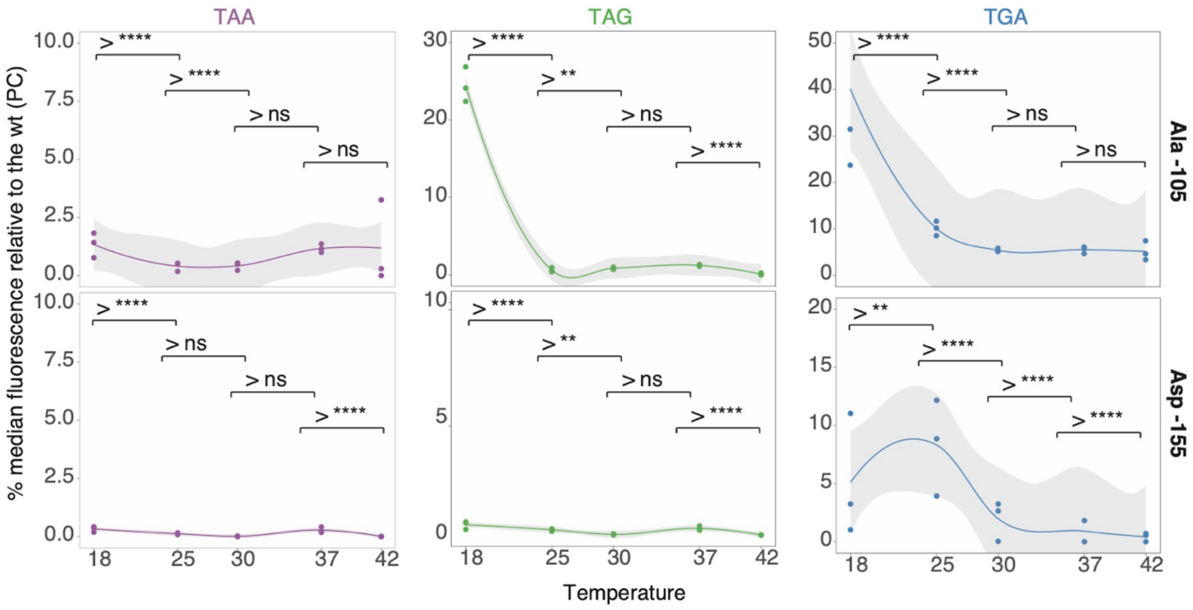

Supplementary Figure 4. Wilcoxon one-sided test shows statistical evidence of a temperature-driven effect on SCR: **18°C>25°C>30°C~37°C>42°C when a stop codon is inserted at positions 105 and 155.** Cell counts were maximized across replicas, excluding those with <200 cells. The test evaluated if one cell distribution significantly exceeded another. Temperature decrease correlates with increased SCR rates, especially notable at higher SCR rates (I.e., at 18°C and with TGA stop codon). Source data is provided as Source data file.

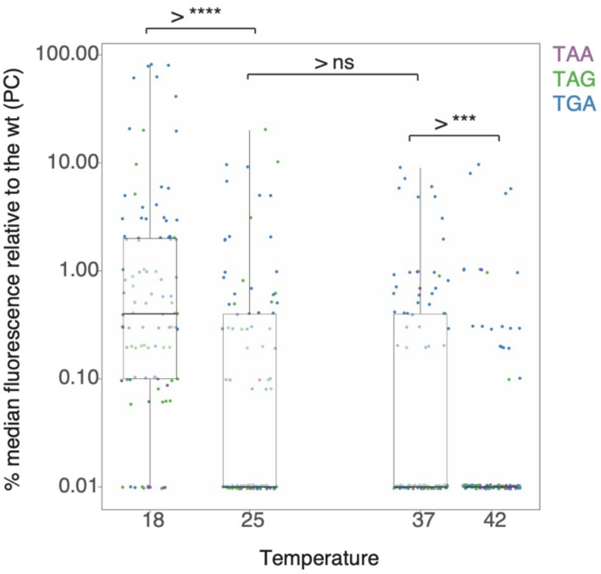

Supplementary Figure 5. Wilcoxon one-sided test shows statistical evidence of a non-linear temperature-driven effect on SCR: 18°C>25°C~37°C>42°C. We assessed the median of fluorescence relative to the wild-type for all the reporters studied in LB and M9 media (dataset from Fig S2), excluding those with no SCR at any of the studied temperatures. Number of datapoint at each temperature = 228, mean at 18°C = 5.50, at 25°C = 0.89, at 37°C = 0.58 and at 42°C = 0.38. Source data is provided as Source data file.

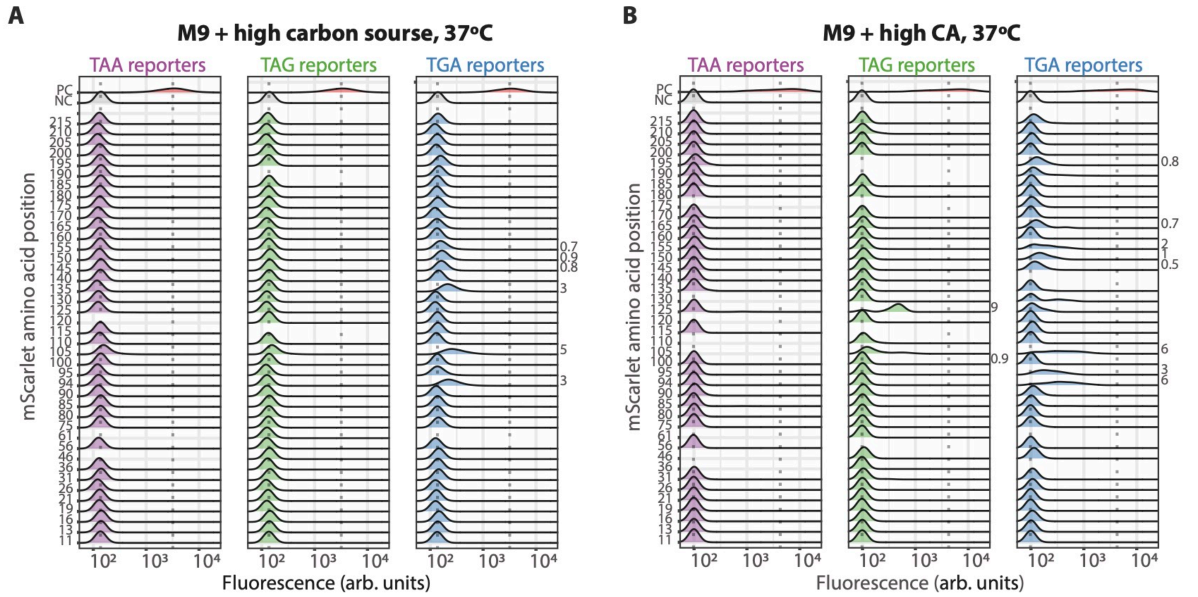

Supplementary Figure 6. Visualization and quantification of stop codon readthrough events in *E. coli* grown in different media. Fluorescence distributions displayed by *E.coli* cells transformed with each library's reporters and grown at 37°C in minimal media supplemented with **A)** high carbon source concentration (M9 media with 1.6% glycerol, 0.2% casamino acids, 1mM thiamine hydrochloride, 2 mM MgSO 4 and 0.1 mM CaCl 2) and **B)** high casamino acid concentration (M9 media with 0.4% glycerol, 0.4% casamino acids, 1mM thiamine hydrochloride, 2 mM MgSO 4 and 0.1 mM CaCl 2). Each distribution is derived from one replicate and 20 to 10967 cells. Source data is provided as Source data file.

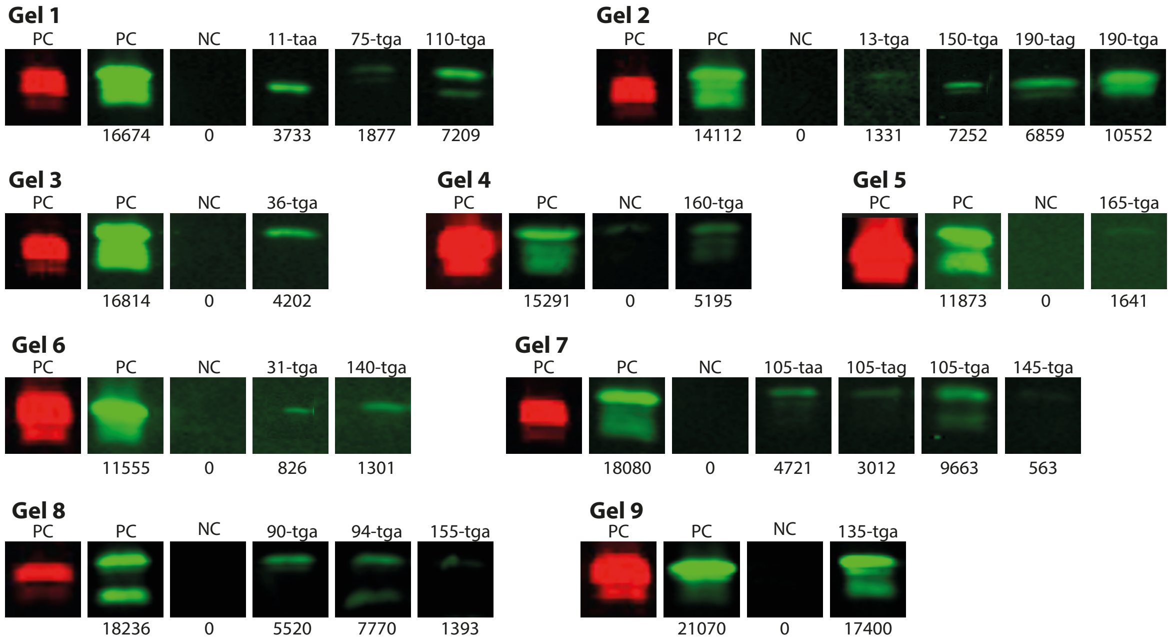

Supplementary Figure 7. Detection of stop codon readthrough by analysing the His-tag expression with His-tag antibodies in bands corresponding to the expected size for full-lengh mScarlet. In-gel fluorescence signals of the PC (cells expressing the mScarlet) are shown in red. The His-tag signal of the positive control (PC), negative control (NC, cells carrying an empty vector), and the cells expressing the reporters are shown in green. The numbers below the images indicate the quantification of the His-tag expression as explained in the Methods section. Cells were grown in rich media at 18°C (see Methods). Full-sized gels are shown in Figure S8.

Gel1

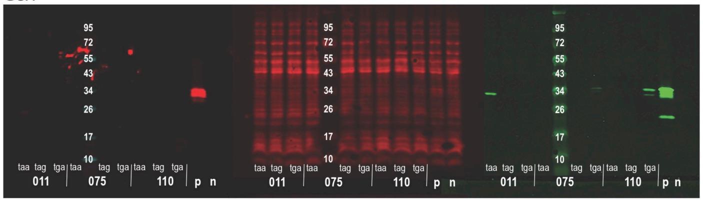

Gel2

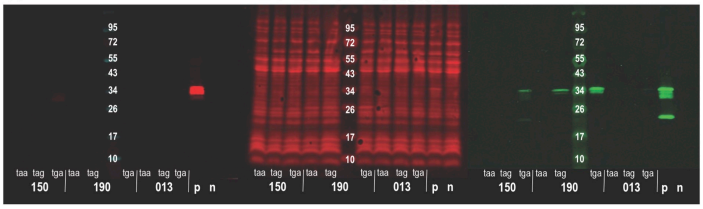

Gel3

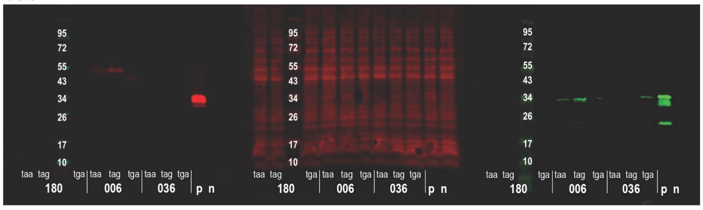

Gel4

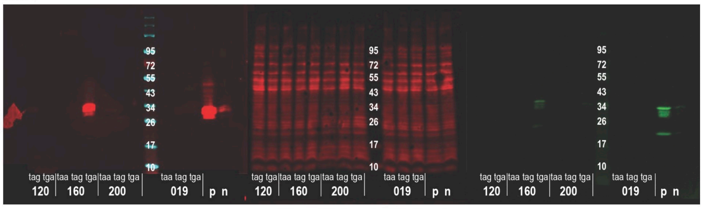

Gel5

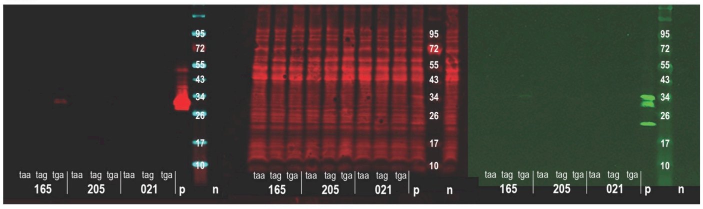

Gel6

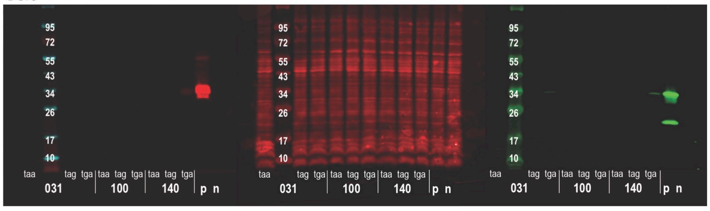

Gel7

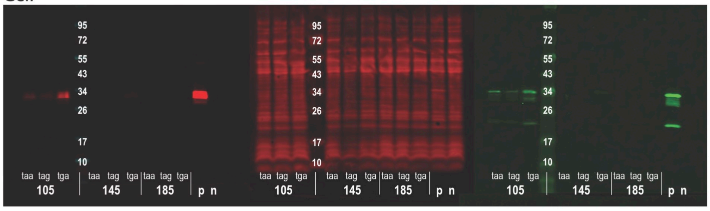

Gel8

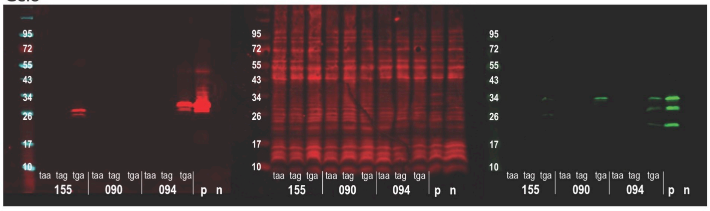

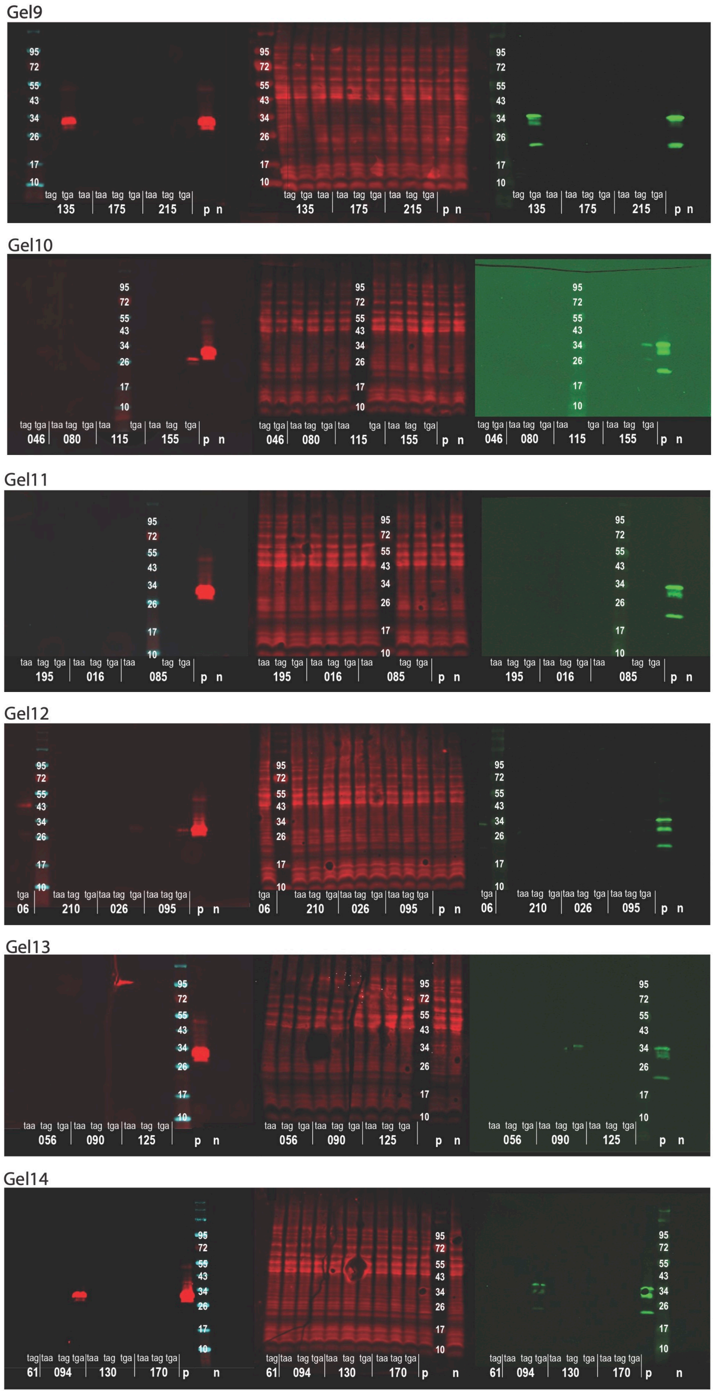

Supplementary Figure 8. Detection of His-tag expression with His-tag antibodies. For each gel it is shown, in the first column the in-gel fluorescence signal, in the middle column, the total protein expression signal assayed using Fast Green FCF staining solution and, in the last column, the his-tag signal. All gels contain the signal of cells expressing the mScarlet wild-type as PC (p) and cells expressing an empty vector as NC (n) together with the protein marker leader. The denomination of the reporter loaded in each well is annotated in the button area of the gel. Source data is provided as Source data file.

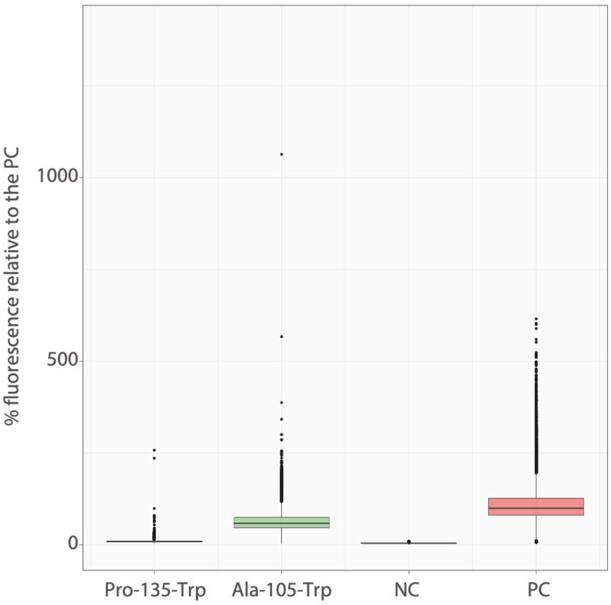

Supplementary Figure 9. While introducing a tryptophan in position 105 did not compromise the fluorescence of the mScarlet, introducing a tryptophan in position 135 decreased the fluorescence of the mScarlet by one order of magnitude. Source data is provided as Source data file.

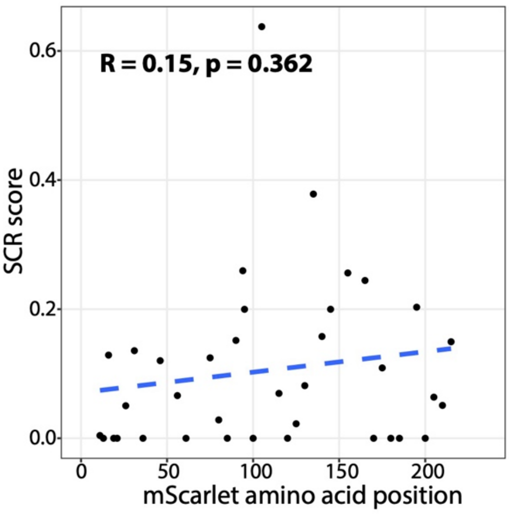

Supplementary Figure 10. Stop codon readthrough events occur evenly along the mScarlet sequence. The SCR score describes an SCR event's likelihood at a given position (see Methods section for further details about the calculation of SCR score). There is no significant correlation between SCR score and mScarlet amino acid position. We excluded four positions with a discordance between His-tag and fluorescence signal over 30% (dark reporters). Source data is provided as Source data file.

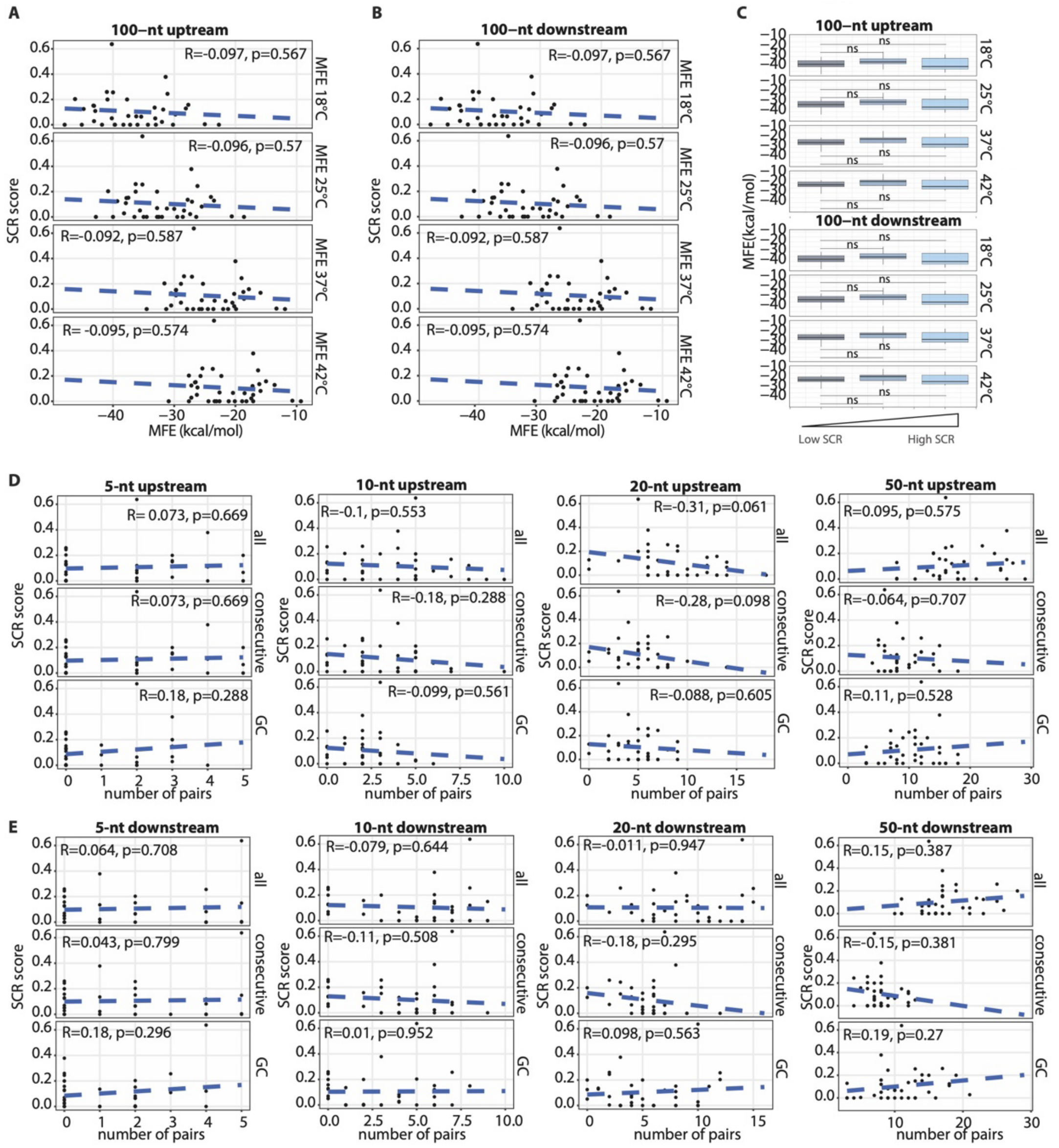

Supplementary Figure 11. The impact of predicted mRNA secondary structure on SCR. A) The predicted minimum free energy (MFE) is shown for the most stable secondary structure elements in a 100-nt window upstream of the premature stop codon at different temperatures. Decreasing the temperature stabilized the predicted RNA secondary structures. There is no correlation between the stability of the predicted RNA secondary structures and the likelihood of SCR (SCR score; see Methods section for further details about the calculation of SCR score). **B)** The predicted minimum free energy (MFE) is shown for the most stable secondary structure elements in a 100-nt window downstream of the premature stop codon at different temperatures. Decreasing the temperature stabilized the predicted RNA secondary structures. However, there is no correlation between the stability of the predicted RNA secondary structures and the likelihood of SCR. **C)** There are no significant differences in the stability (MFE) of the predicted RNA secondary structures of the reporters with no SCR errors, mid-tendency to SCR errors, and high-tendency to SCR errors (see Methods section for further details about the binning into three categories of the SCR score). **D)** The local thermodynamic stability of the secondary structures (i.e., the number of all base pairs, G-C pairs, and the longest stretch of consecutive pairs) in a set of 5, 10, 20, and 50-nt windows upstream of the premature stop codon does not correlate with the likelihood of SCR events. **E)** The local thermodynamic stability of the secondary structures (i.e., the number of all base pairs, G-C pairs, and the longest stretch of consecutive pairs) in a set of 5, 10, 20, and 50-nt windows downstream of the premature stop codon does not correlate with the likelihood of SCR events. Source data is provided as Source data file.

Ala-105-TAA

(R)VMNFEDGGAVTVTQDTSLEDGTLIYK(V)

sissi_rep_3_5_100ul_01.11519.11519.2.dta

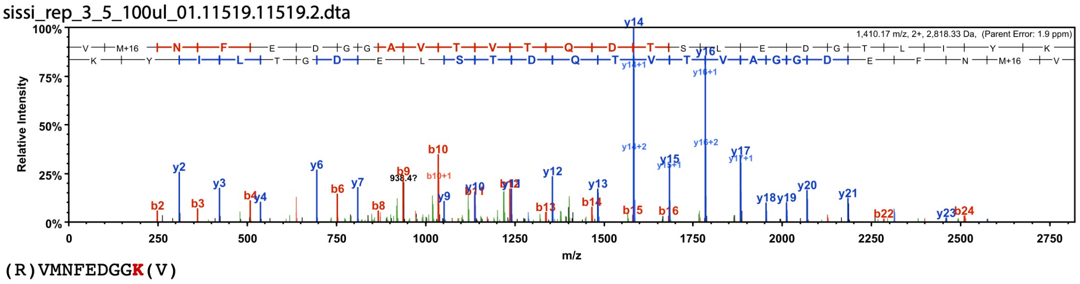

(R)VMNFEDGGK(V)

sissi_5_rep3_100ul.02204.02204.2.dta

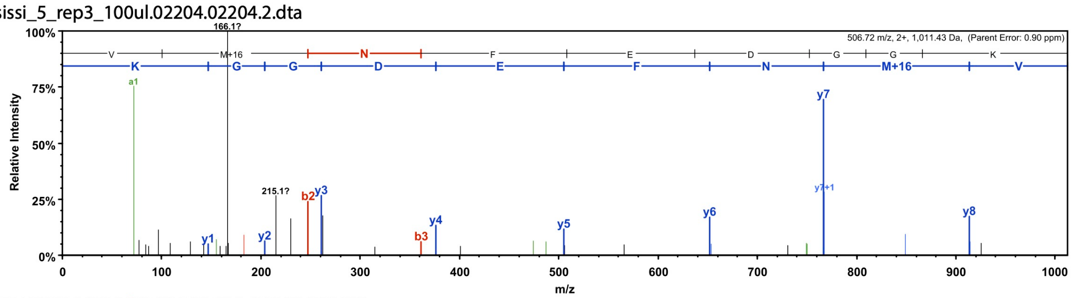

(R)VMNFEDGGQVTVTQDTSLEDGTLIYK(V)

sissi_rep_3_5_100ul_01.10879.10879.2.dta

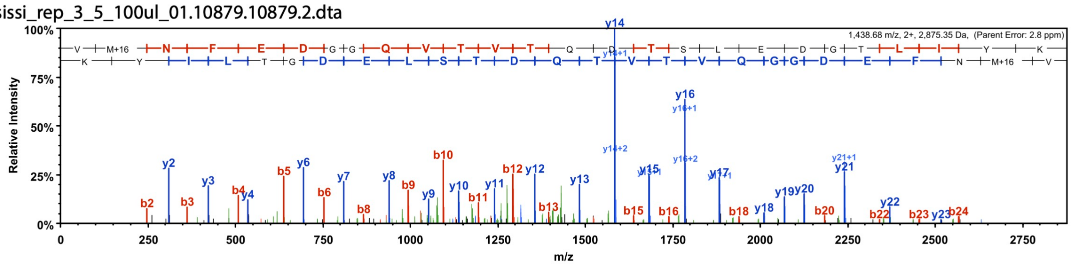

(R)VMNFEDGGSVTVTQDTSLEDGTLIYK(V)

s_s5_rep3_100ul_01.05091.05091.2.dta

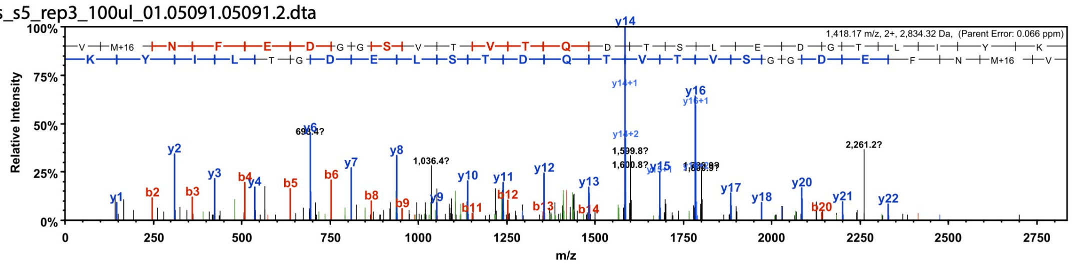

(R)VMNFEDGGYVTVTQDTSLEDGTLIYK(V)

sissi_rep_3_5_100ul_01.12077.12077.2.dta

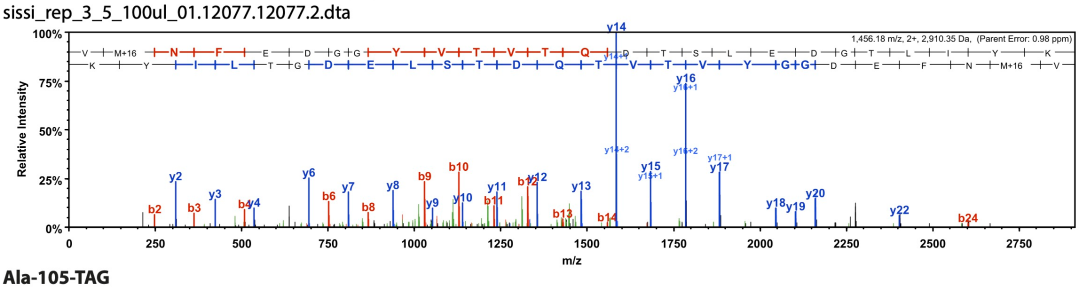

Ala-105-TAG

(R)VMNFEDGGAVTVTQDTSLEDGTLIYK(V)

sissi_rep_3_6_100ul_01.11857.11857.2.dta

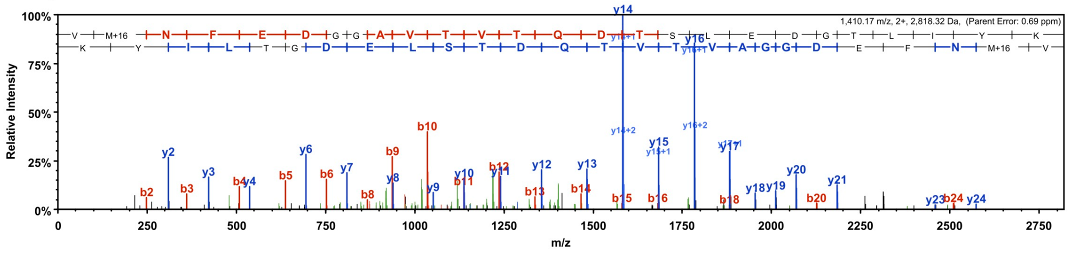

(R)VMNFEDGG**K**(V)

sissi_6_rep3_100ul.02714.02714.2.dta

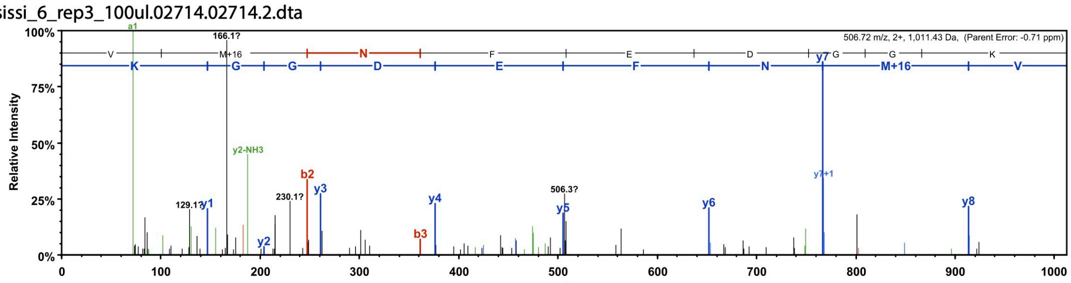

(R)VMNFEDGG**Q**VTVTQDTSLEDGTLIYK(V)

sissi_rep_3_6_100ul_01.13331.13331.2.dta

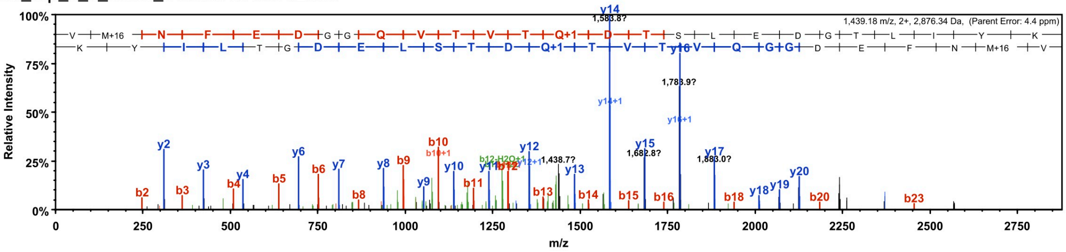

(R)VMNFEDGG**S**VTVTQDTSLEDGTLIYK(V)

sissi_rep_3_6_100ul_01.11857.11857.2.dta

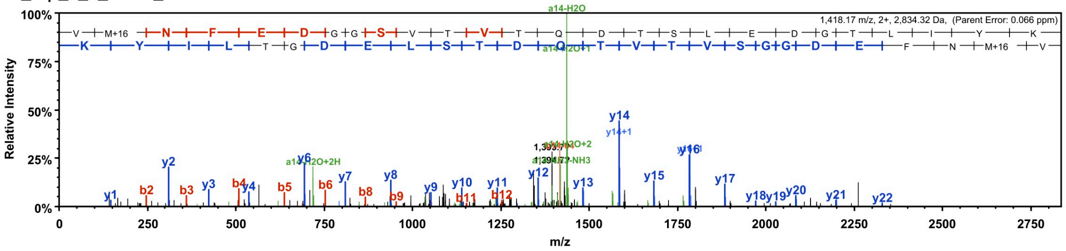

(R)VMNFEDGG**W**VTVTQDTSLEDGTLIYK(V)

s_s6_rep3_100ul_01.06082.06082.2.dta

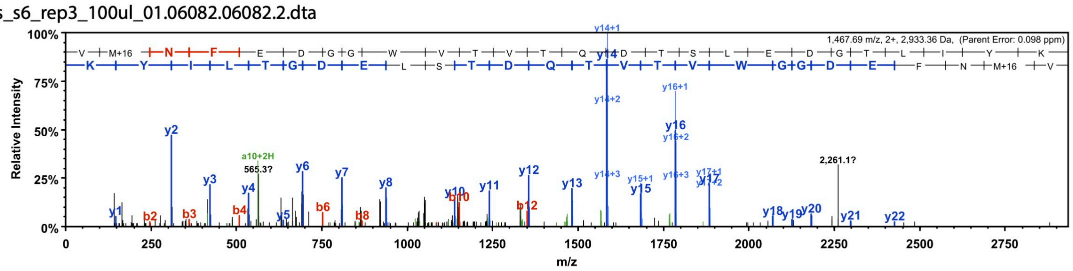

(R)VMNFEDGG**Y**VTVTQDTSLEDGTLIYK(V)

sissi_rep_3_6_100ul_01.12443.12443.2.dta

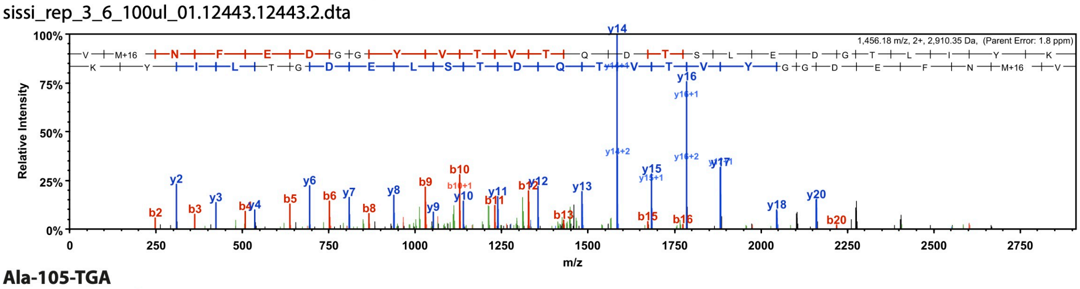

Ala-105-TGA

(R)VMNFEDGG**C**VTVTQDTSLEDGTLIYK(V)

s3_lane9_50ul.43349.43349.2.dta

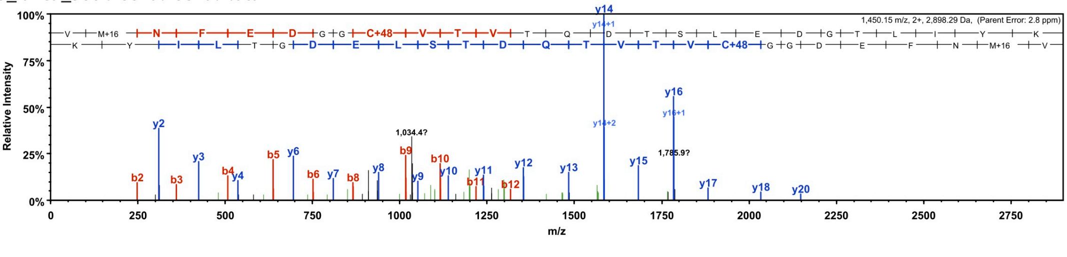

(R)VMNFEDGGWVTVTQDTSLEDGTLIYK(V)
s3_lane9_50ul.75236.75236.2.dta

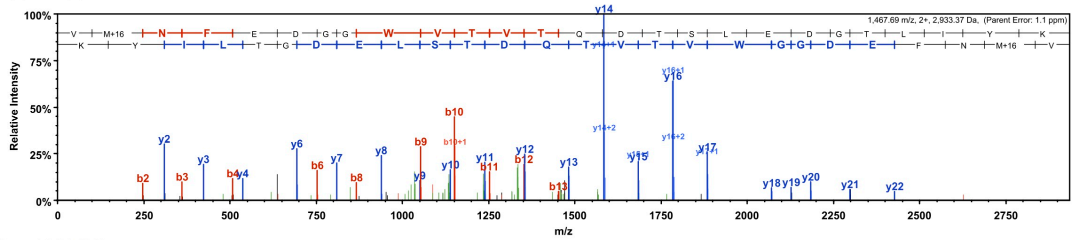

Pro-135-TAG

(K)LRGTNFPPDGK(V)
s2_lane3_11_50ul_210629211931.09651.09651.2.dta

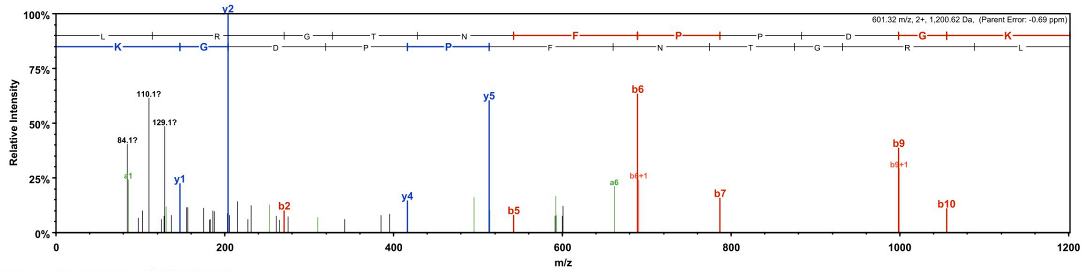

(K)LRGTNFPPDGQVMQK(K)
s2_lane3_11_50ul_210629211931.14799.14799.2.dta

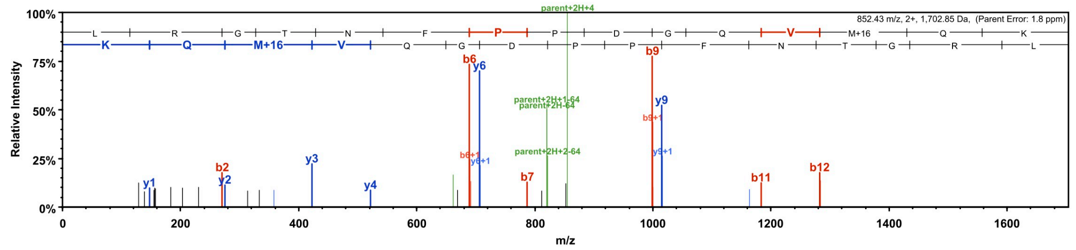

(K)LRGTNFPPDGYVMQK(K)
s2_lane3_11_50ul_210629211931.20047.20047.2.dta

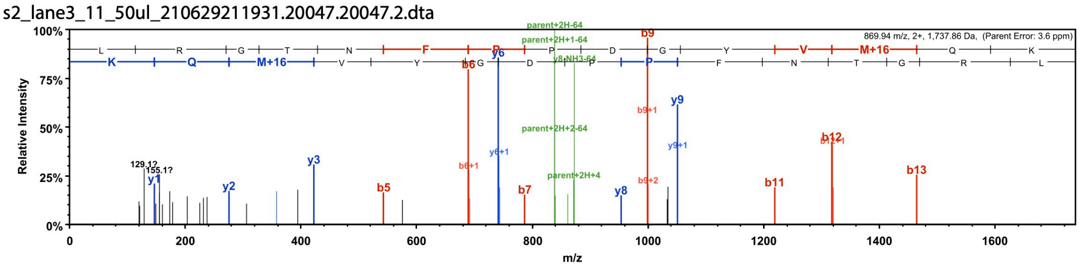

Pro-135-TGA

(K)LRGTNFPPDGCVMQK(K)
s1_lane9_50ul.15076.15076.2.dta

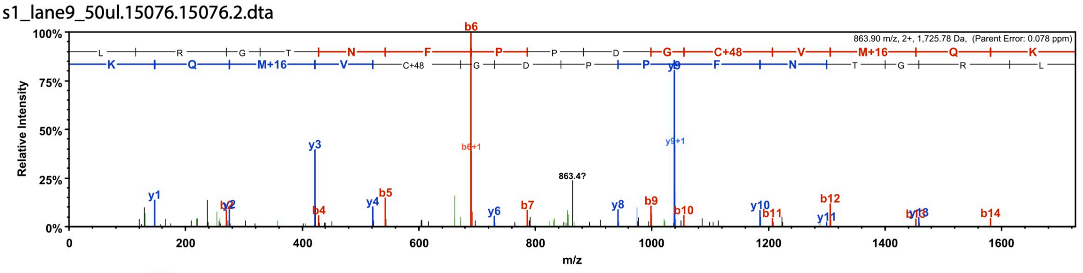

(K)LRGTNFPPDGWVMQK(K)
s1_lane9_50ul.18960.18960.2.dta

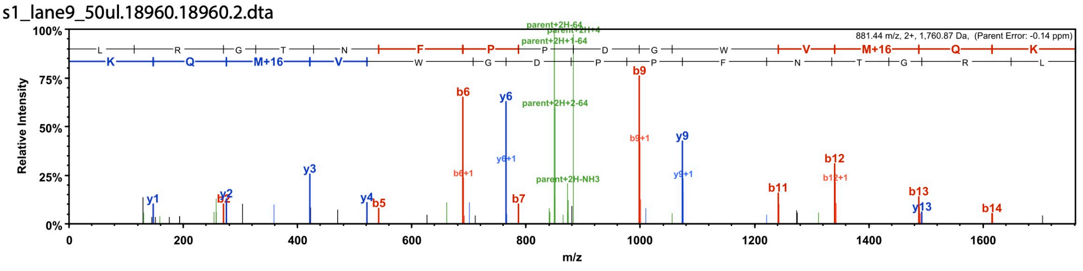

Glu-145-TAG

(K)TmGWQASTER(L)

s_8_2.03361.03361.2.dta

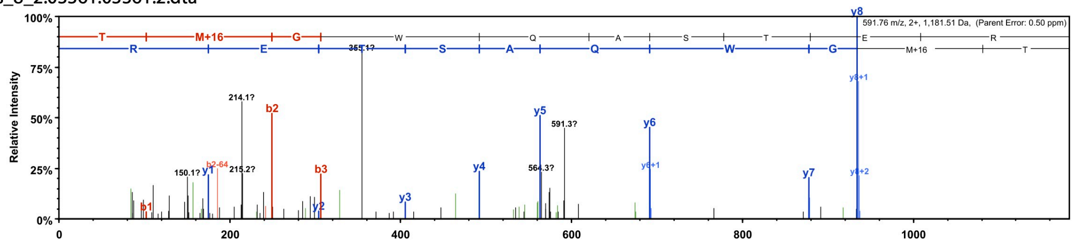

(K)TmGWWASTER(L)

s_8_2.06897.06897.2.dta

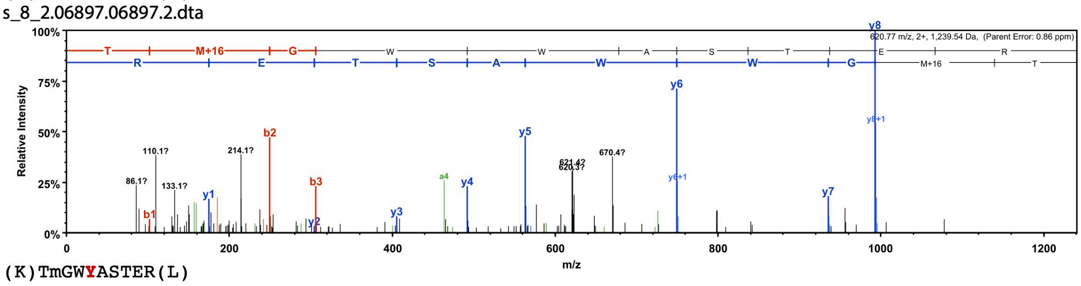

(K)TmGWYASTER(L)

s_8_2.04175.04175.2.dta

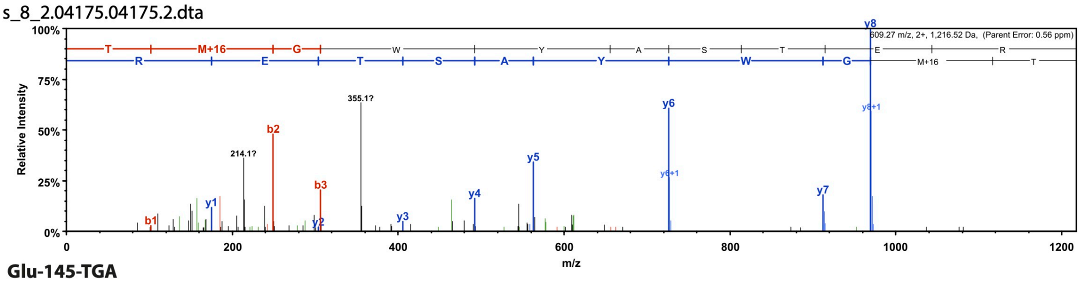

Glu-145-TGA

(K)TMGWEASTER(L)

s_s7_rep3_100ul_01.02210.02210.2.dta

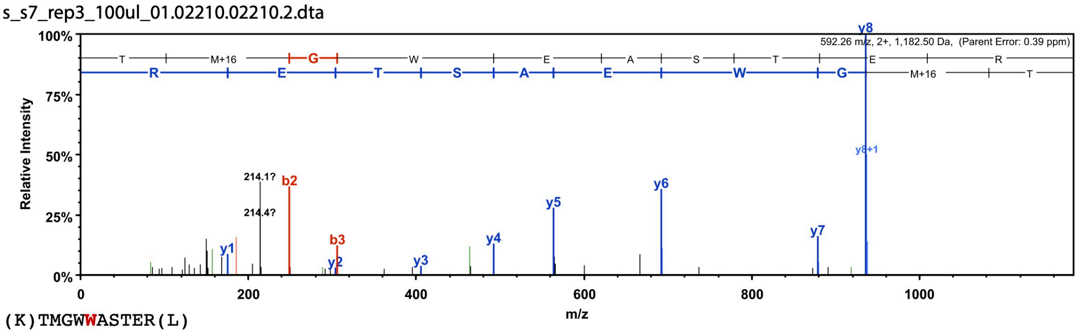

(K)TMGWWASTER(L)

sissi_7_rep3_100ul.12372.12372.2.dta

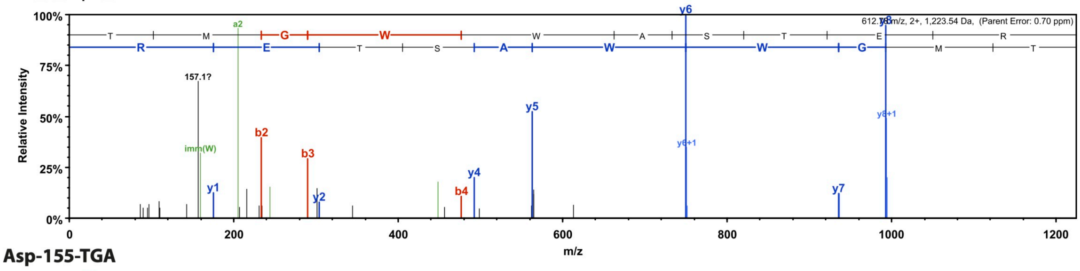

Asp-155-TGA

(R)LYPEDGVLK(G)

s_s4_2_100ul_02.02942.02942.2.dta

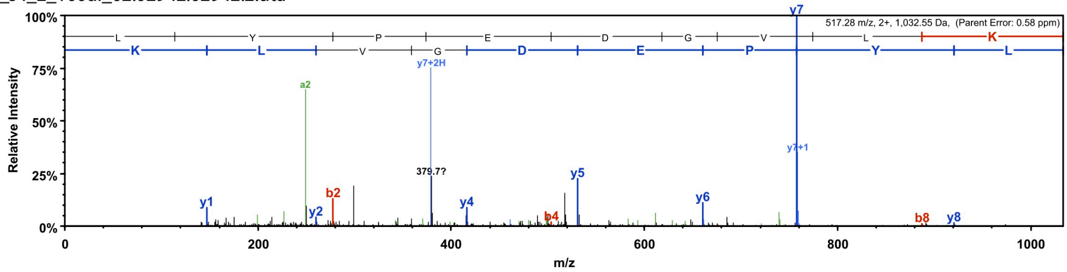

(R)LYPEWGVLK(G)
4_50ul_2.15909.15909.2.dta

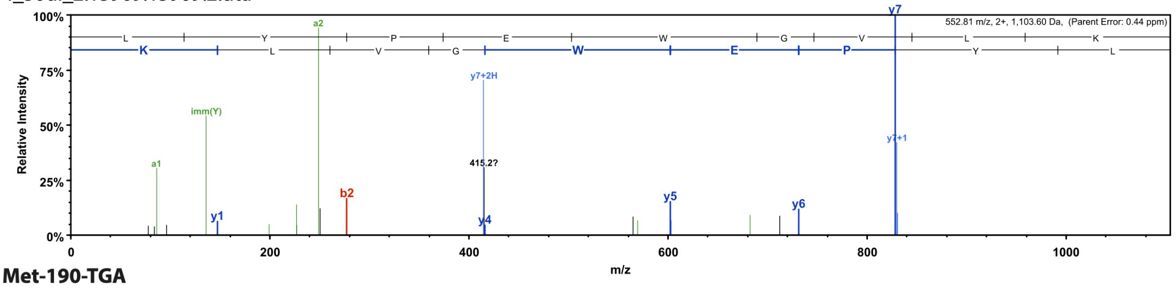

Met-190-TGA
(K)KPVQCPGAYNVDR(K)
m190_1_20220125103726.03059.03059.2.dta

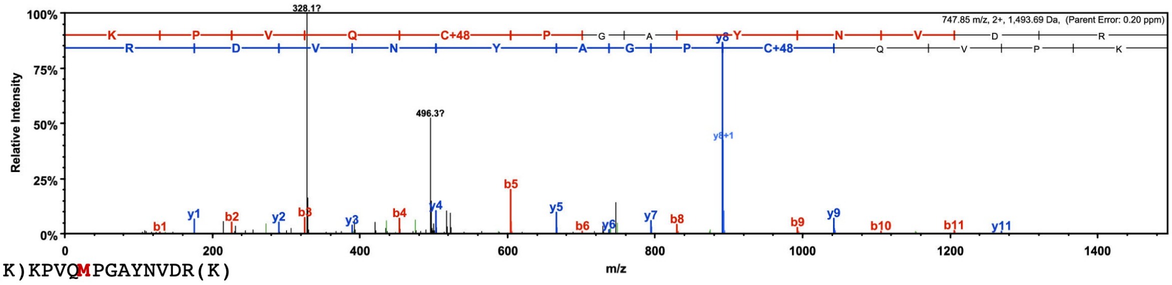

(K)KPVQMPGAYNVDR(K)
s_m190_100ul_01.02587.02587.3.dta

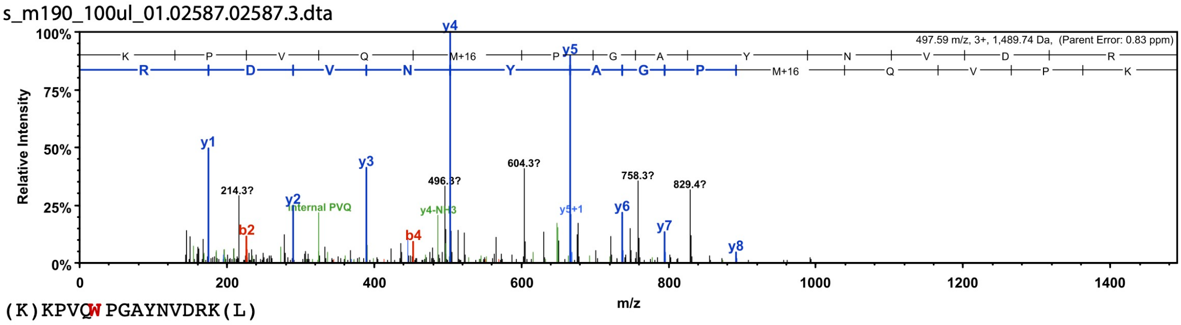

(K)KPVQWPGAYNVDRK(L)
m190 1 20220125103726.06315.06315.2.dta

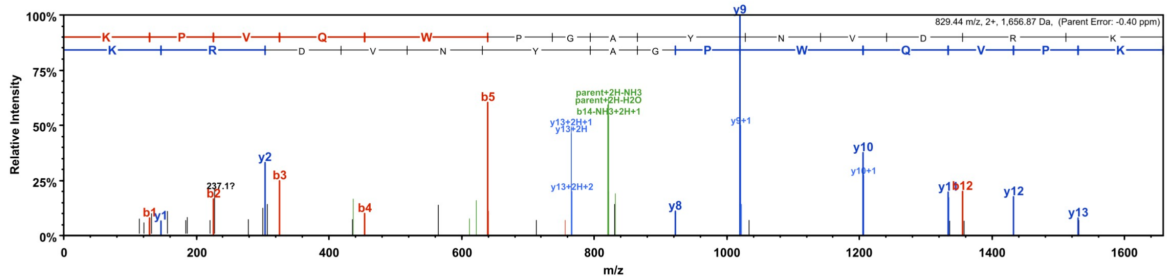

Supplementary Figure 12. Representative MS-spectra of reporters reveal the amino acids misincorporated at premature stop codon sites. We selected 10 reporters for targeted mass spectrometry analysis. The spectra correspond to the peptides that cover the stop codon site. The amino acid that was inserted at the stop codon site is highlighted in red (see Supplementary Table S4 and Methods section for quantification of amino acid misincorporations).

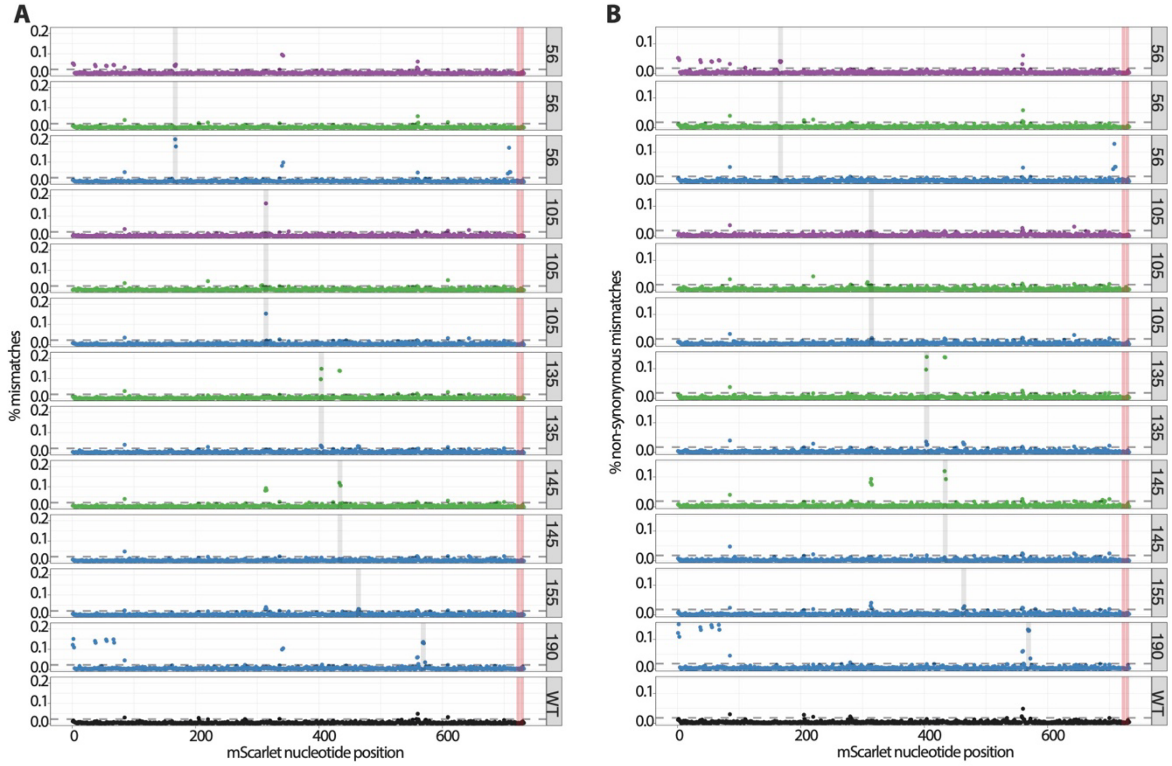

Supplementary Figure 13. RNA polymerase errors at premature stop codons revealed by RNA-seq data. The percentages of nucleotide mismatches are shown along the mScarlet mRNA sequence for selected reporters. While most positions have a very low error rate, a few positions have a higher percentage of mismatches (A) and mismatches that result in an amino acid change (B, non-synonymous mismatches). The premature stop codon is indicated with grey shades and the canonical stop codon at the C-terminal is highlighted in red. There is no difference between reporters that harbor different stop codons, TAA (purple), TAG (green), and TGA (blue). The wild-type sequence is shown in black. Raw RNA-sew data is provided as Data S2. Source data is provided as Source data file.

AS**W**KPLLNLLP (2+, m/z = 626.37744)

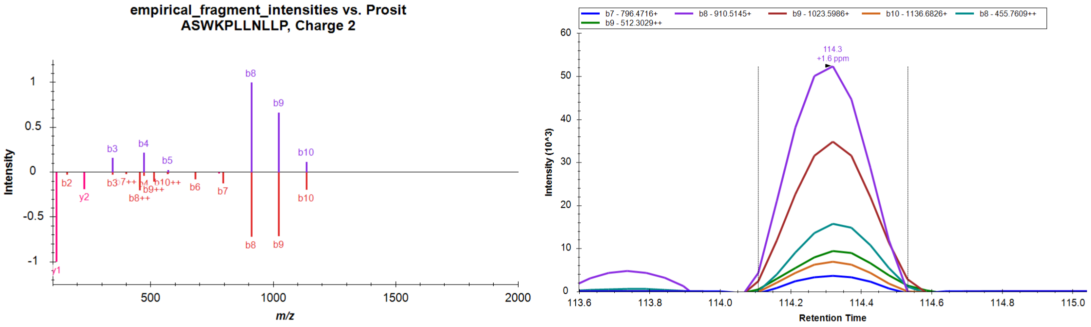

DDEAEK**T**EINGVAK (2+, m/z = 759.86859)

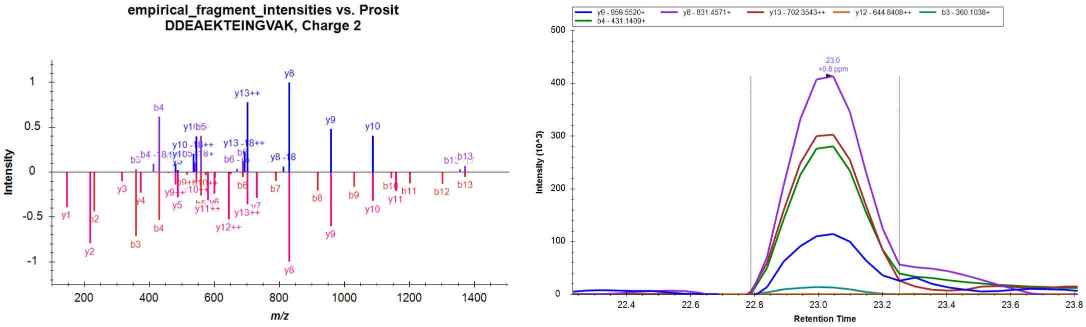

EITMGKTQPLPILITGGGR (3+, m/z = 666.70734)

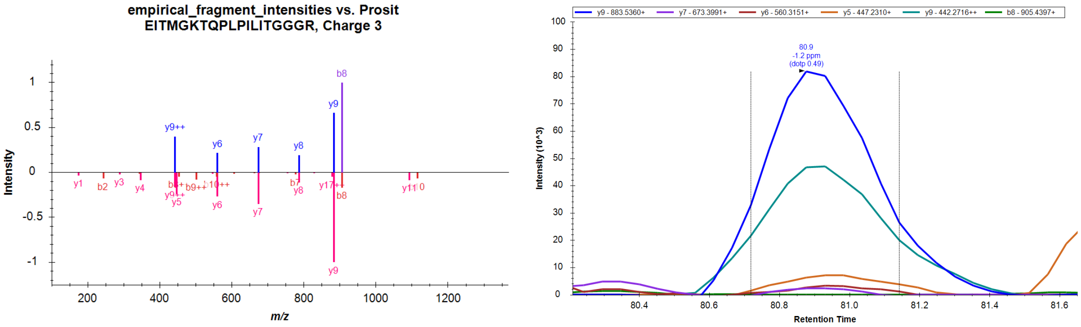

GPAAVNVTAI**W**SNPLI (2+, m/z = 811.94952)

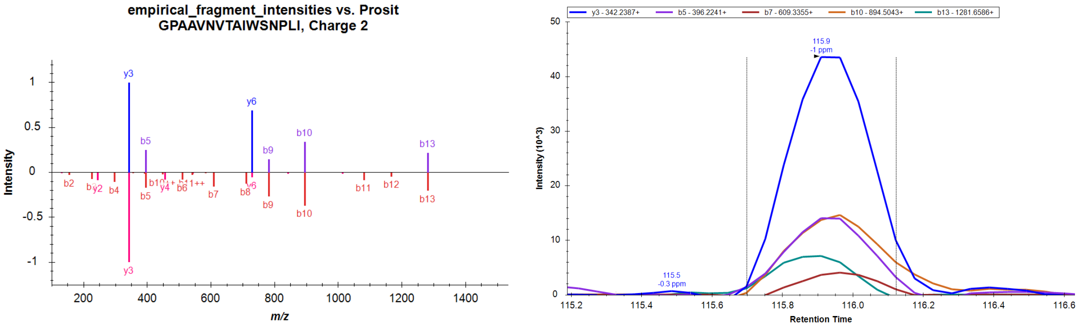

GSS**W**WSSVPLSDQMSR (2+, m/z = 905.41571)

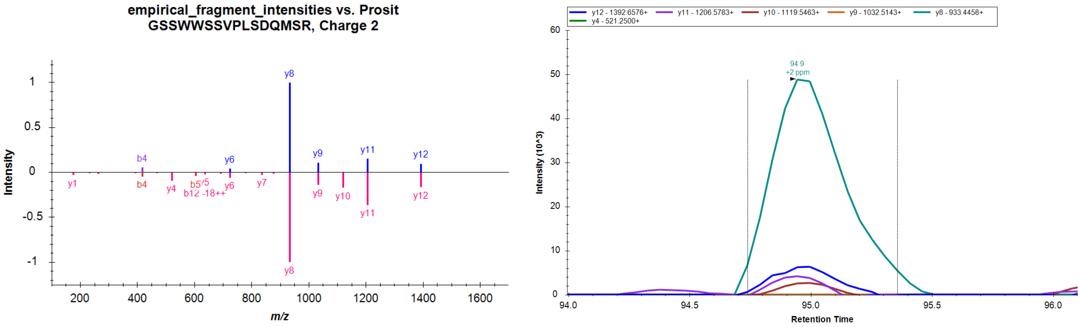

GVFAPLQII<u>C</u>VV (2+, m/z = 658.37659)

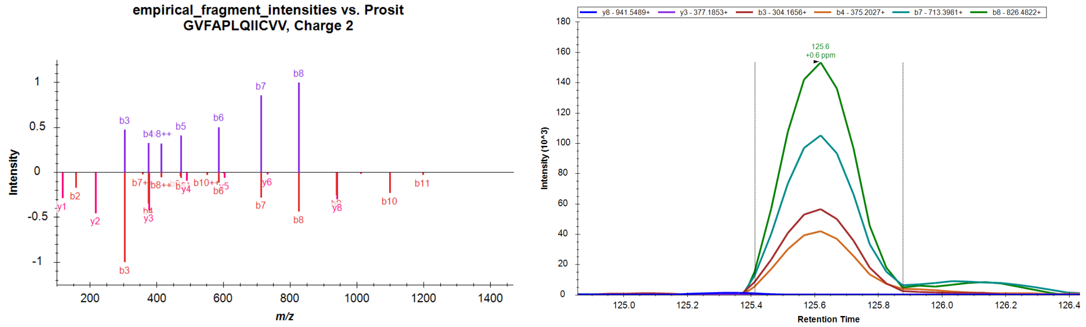

ILIS**F**IR (2+, m/z = 431.28229)

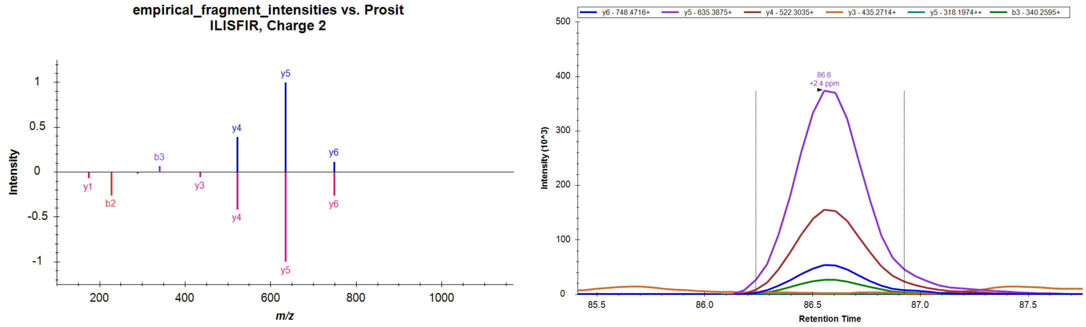

KAGEAA**V**TVK (2+, m/z = 487.2883)

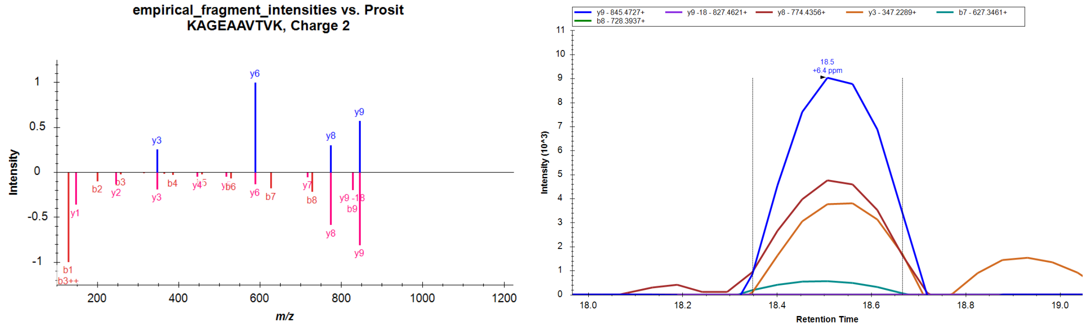

KVA**P**GQNIASSR (2+, m/z = 614.34467)

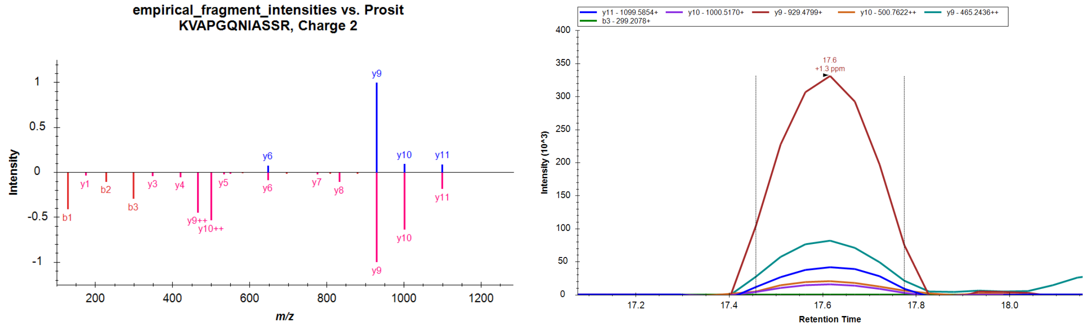

LAHQAMTLK (2+, m/z = 506.78488)

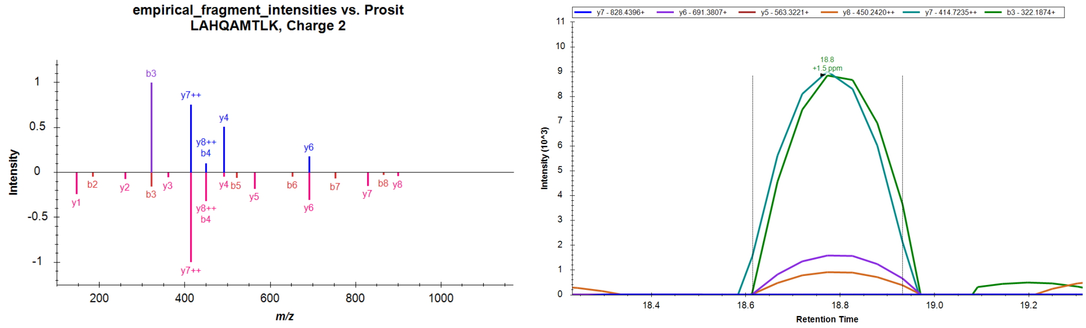

NIAATLAIGMRNAGMQGR (2+, m/z = 930.98102)

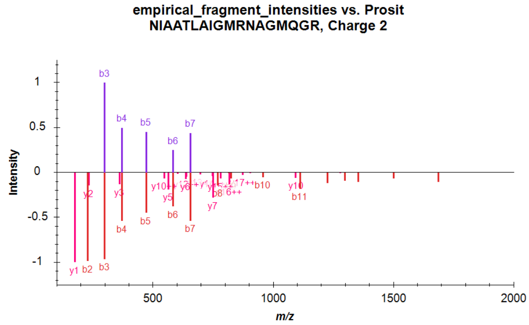

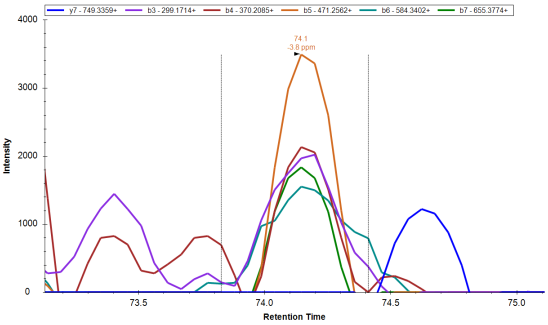

QPALGYLNCTPK (2+, m/z = 681.34857)

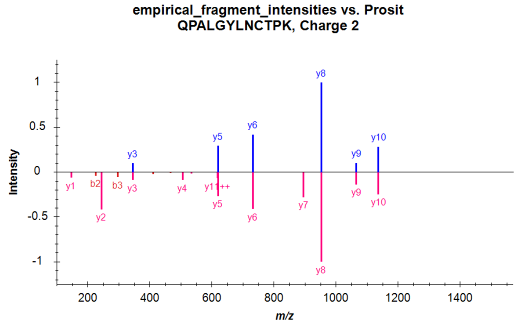

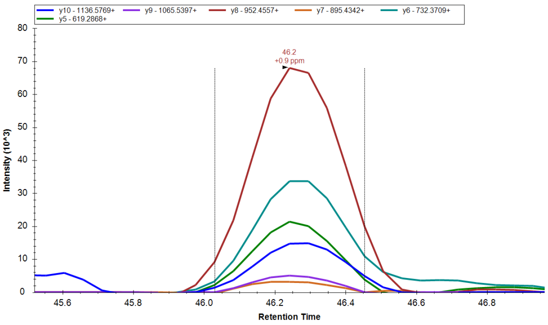

QPALGYLN**W**TPK (2+, m/z = 681.34857)

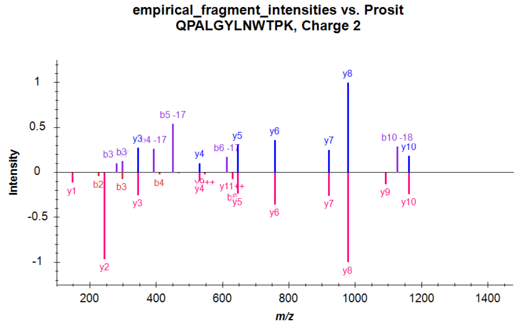

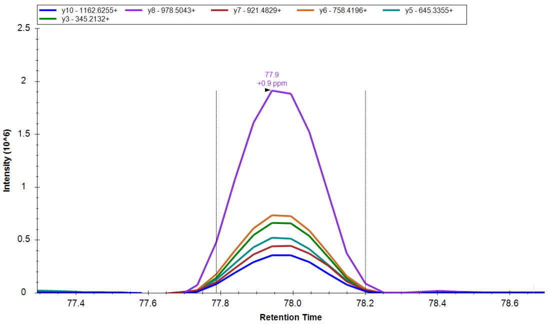

SEDAMSTQLDPTQLAIEFLR (4+, m/z = 567.03284)

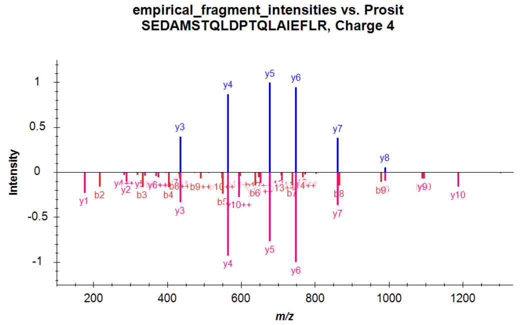

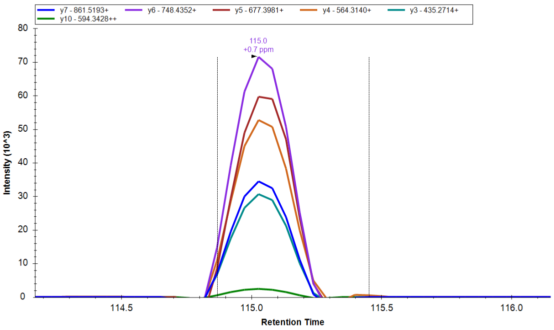

VLLIP**W**NR (2+, m/z = 505.81174)

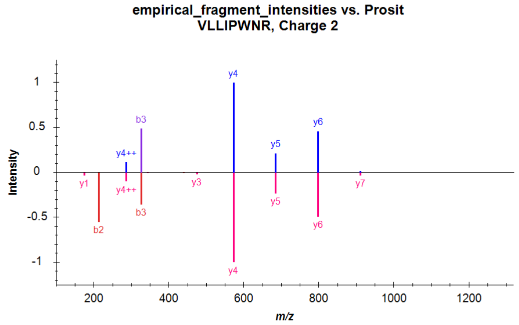

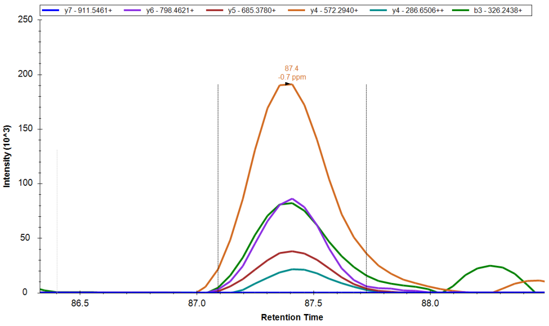

VYAGNEHNHAAQQPQVLDICSGL (3+, m/z = 841.06854)

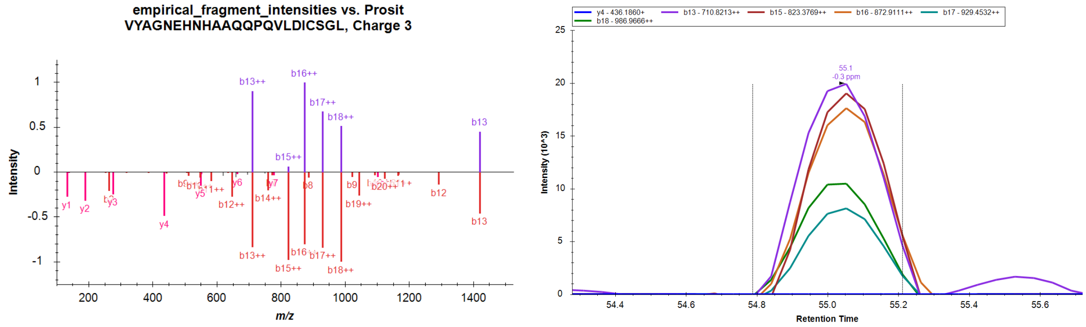

Supplementary Figure 14. Evidence for peptides in Table S5 showing stop codon readthrough in the *E. coli* proteome. The amino acid inserted at the Stop codon is shown in **bold**. If no amino acid is bold, the peptide is completely downstream of the Stop codon. Underlined residues are modified: carbamidomethylation (+57) at C, oxidation (+18) at M. The data shown represent the evidence used by DIA-NN to identify the peptides. For each peptide, the left panel shows a mirror plot of empirical relative fragment intensities extracted by DIA-NN as they appear in the output spectral library (upper spectrum), compared to Prosit prediction (lower spectrum; Prosit spectra predicted with NCE 31, taking into account the offset of 6 between Prosit and Orbitrap NCE[1]). Note that DIA-NN does not take into account the ions b1, b2, y1, and y2, even when predicted to be intense, due to a high likelihood of interference. For this reason, the dot product calculated by Skyline is not applicable and not shown. The right panels show chromatograms (with Savitsky-Golay smoothing) of fragments in the output spectral library from a single replicate, with peak boundaries as reported in the DIA-NN main output table, and mass error (in ppm) for the displayed replicate as calculated by Skyline.

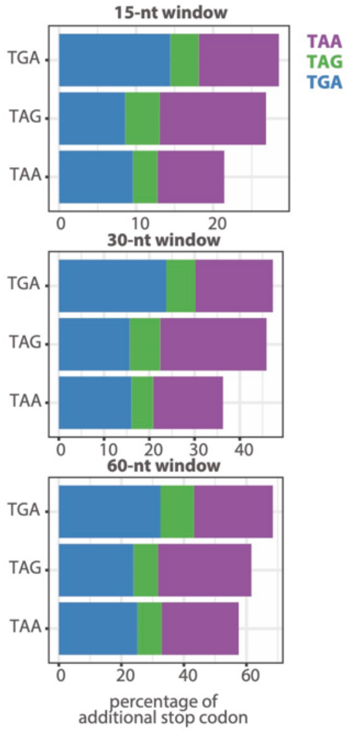

Supplementary Figure 15. The occurrence of an additional stop codon in a 15, 30, and 60-nt window downstream of the stop codon correlates with the protein synthesis accuracy of the stop codon in the *E. coli* genome of the BL21 strain. TGA, as the least accurate stop codon, has the highest frequency of an additional stop codon in the 3' regions of genes. Source data is provided as Source data file.

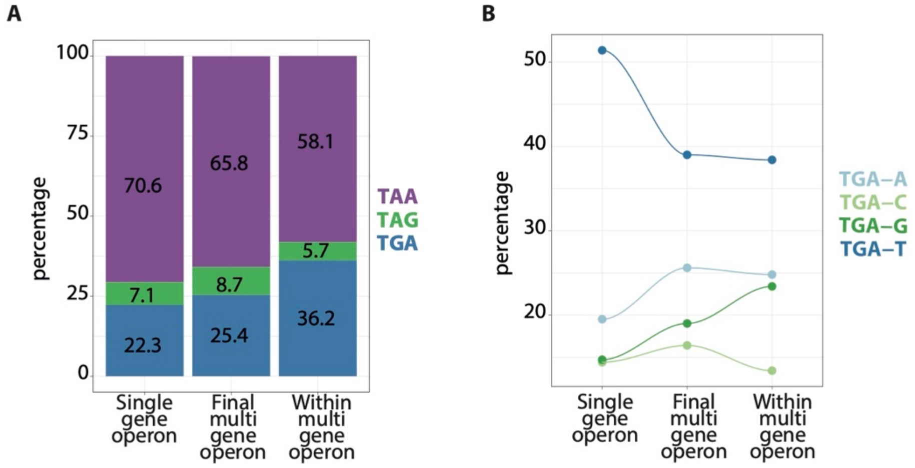

Supplementary Figure 16. Stop codon readthrough events may be more likely among genes within multi-operons. Using the RegulonDB database[2], we classified the *E. coli* genes into three categories: 1) *Single gene operons*, those in operons that contain only one gene. 2) *Final multi-gene operons*, those expressed last within the multi-gene operon. 3) *Within multi-gene operons*, those not expressed last within the multi-gene operon. **A)** Genes within multi-operon are enriched in TGA, the most error-prone codon, while depleted in TAA, the most accurate, compared to genes that belong to single-gene and final multi-gene operons. **B)** Genes within multi-operon are enriched in G and depleted in T at the first nucleotide position downstream of the TGA stop codon. We found that the presence of T increases and G decreases the protein synthesis termination efficiency (Fig 3B and 3C). Source data is provided as Source data file.

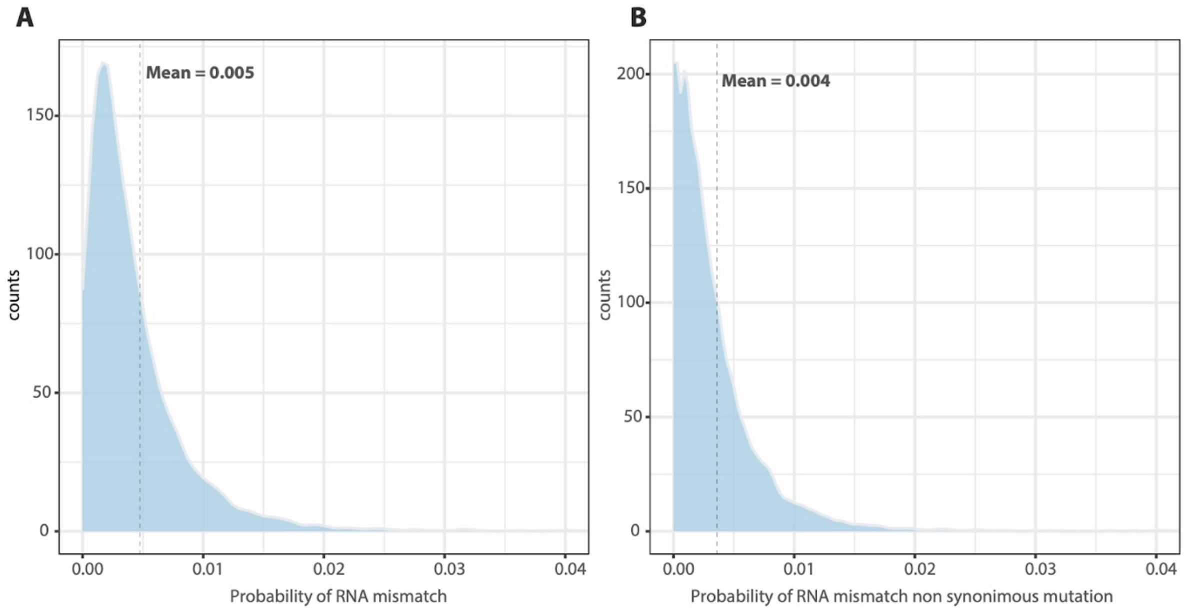

Supplementary Figure 17. Empirical calculation of RNA nucleotide misincorporation error rate (percentage of mRNA nucleotide mismatch per base) for **A)** all types of RNA mismatches and **B)** non-synonymous RNA mismatches. Source data is provided as Source data file.

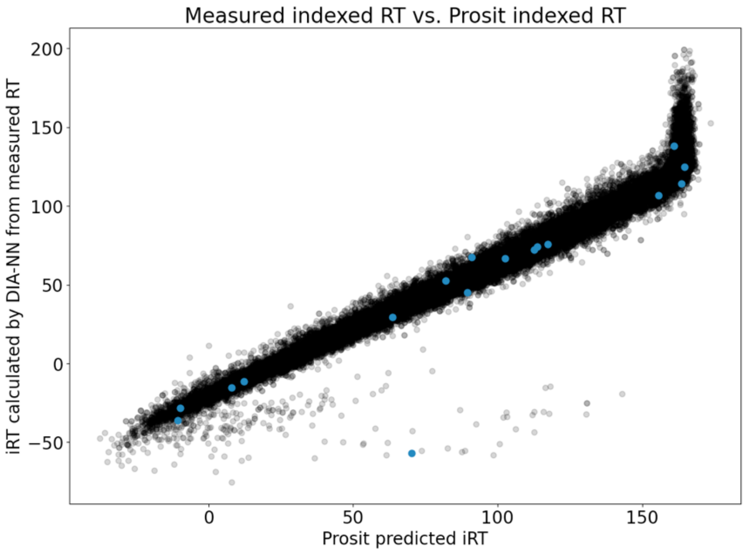

Supplementary Figure 18. Agreement between indexed retention times predicted by Prosit[3] and indexed RT calculated from measured RT. For all precursors reported by DIA-NN, indexed retention times were predicted with Prosit. The plot shows the iRT predicted by Prosit vs. the iRT calculated by DIA-NN, as given in its main output table. Each dot represents a precursor, SCR precursors are shown in blue. The outlier SCR precursor in the bottom right part, eluting much earlier than predicted, was removed from the analysis. Source data is provided as Source data file.

Supplementary Table 1. Friedman (when comparing 3 replicas) and sign (when comparing 2 replicas) two-sided testing confirm a high level of cell-to-cell variability, indicating significant differences among biological replicates. Replicas with fewer than 200 cells were excluded from the analysis. Empty cells represent instances where only one replica displayed more than 200 cells and the test could not be performed. Source data is provided as Source data file.

Position	Stop codon	T (°C)	Friedman chi-squared	Degree of freedom	p-value
105	TAA	18	832.49	2	< 2.2e-16
105	TAG	18	52.338	2	4.315e-12
105	TGA	18	2277.7	2	< 2.2e-16
105	TAA	25	0.31694	1	0.5734
105	TAG	25	42.497	2	5.914e-10
105	TGA	25	72.741	2	< 2.2e-16
105	TAA	30	21.835	2	1.813e-05
105	TAG	30	70.029	2	6.215e-16
105	TGA	30	72.824	1	< 2.2e-16
105	TAA	37	18.638	1	1.58e-05
105	TAG	37	31.358	2	1.551e-07
105	TGA	37	25.535	2	2.851e-06
105	TAA	42	1927.5	2	< 2.2e-16
105	TAG	42	113.33	2	< 2.2e-16
105	TGA	42	436.02	2	< 2.2e-16
155	TAA	18	1311.9	2	< 2.2e-16
155	TAG	18	441.7	2	< 2.2e-16
155	TGA	18	943.45	1	< 2.2e-16
155	TAA	25	7.4871	2	0.02367
155	TAG	25	41.11	2	1.183e-09
155	TGA	25	236.3	1	< 2.2e-16
155	TAA	30	-	0	-
155	TAG	30	2.4378	1	0.1184
155	TGA	30	16.124	1	5.932e-05
155	TAA	37	51.265	2	7.38e-12
155	TAG	37	30.643	2	2.218e-07
155	TGA	37	-	0	-
155	TAA	42	295.2	2	< 2.2e-16
155	TAG	42	53.465	2	2.456e-12
155	TGA	42	199.69	2	< 2.2e-16

Supplementary Table 2. Wilcoxon one-sided testing, adjusted using the Bonferroni method, shows statistical evidence of a temperature-driven effect on SCR, analysing the highest cell counts across replicas and excluding those with fewer than 200 cells. The test assessed whether one cell distribution was significantly greater than another. Temperature decrease leads to higher SCR rates in a non-linear fashion, especially notable at 18°C. This effect is more pronounced with higher SCR rates and may be less apparent at low SCR rates. Source data is provided as Source data file.

**Ala-105-TGA**	**n1**	**n2**	**statistic**	**p-value**	**p-value.adj**	**p.adj.signif**
18>25	1188	1188	612277	1.05E-106	1.05E-105	****
18>30	1188	1188	682781	3.06E-171	3.06E-170	****
18>37	1188	1188	691100	7.14E-180	7.14E-179	****
18>42	1188	1188	657809	1.28E-146	1.28E-145	****
25>30	1188	1188	541192	3.19E-57	3.19E-56	****
25>37	1188	1188	553799	7.4E-65	7.4E-64	****
25>42	1188	1188	485879	1.57E-29	1.57E-28	****
30>37	1188	1188	361295	0.245	1	ns
30>42	1188	1188	328111	0.983	1	ns
37>42	1188	1188	312479	1	1	ns
**Asp-155-TGA**	**n1**	**n2**	**statistic**	**p-value**	**p-value.adj**	**p.adj.signif**
18>25	731	731	153135	0.000349	0.003	**
18>30	731	731	188044	1.03E-21	1.03E-20	****
18>37	731	731	205217	3.32E-36	3.32E-35	****
18>42	731	731	238965	4.68E-76	4.68E-75	****
25>30	731	731	209597	1.59E-40	1.59E-39	****
25>37	731	731	231594	4.61E-66	4.61E-65	****
25>42	731	731	248095	1.96E-89	1.96E-88	****
30>37	731	731	186012	2.94E-20	2.94E-19	****
30>42	731	731	229364	3.51E-63	3.51E-62	****
37>42	731	731	204646	1.16E-35	1.16E-34	****
**Ala-105-TAG**	**n1**	**n2**	**statistic**	**p-value**	**p-value.adj**	**p.adj.signif**
18>25	3150	3150	4762079	0	0	****
18>30	3150	3150	4946163	0	0	****
18>37	3150	3150	4949600	0	0	****
18>42	3150	3150	4947129	0	0	****
25>30	3150	3150	2658833	0.000255	0.003	**
25>37	3150	3150	2374101	0.982	1	ns
25>42	3150	3150	3624003	2.9E-111	2.9E-110	****
30>37	3150	3150	1904772.5	1	1	ns
30>42	3150	3150	3433016	7.42E-78	7.42E-77	****
37>42	3150	3150	3633638	4.14E-113	4.14E-112	****
**Asp-155-TAG**	**n1**	**n2**	**statistic**	**p-value**	**p-value.adj**	**p.adj.signif**
18>25	434	434	70856	7.24E-20	7.24E-19	****
18>30	434	434	77709	9.09E-32	9.09E-31	****
18>37	434	434	59317	1.78E-06	1.78E-05	****
18>42	434	434	80741	5.61E-38	5.61E-37	****
25>30	434	434	55492	0.000756	0.008	**
25>37	434	434	37662	1	1	ns
25>42	434	434	76355	3.5E-29	3.5E-28	****
30>37	434	434	32807	1	1	ns
30>42	434	434	75328	2.68E-27	2.68E-26	****
37>42	434	434	77743	7.8E-32	7.8E-31	****
**Ala-105-TAA**	**n1**	**n2**	**statistic**	**p-value**	**p-value.adj**	**p.adj.signif**
18>25	729	729	177737	1.95E-15	1.95E-14	****
18>30	729	729	220320	1.93E-53	1.93E-52	****
18>37	729	729	177688	2.09E-15	2.09E-14	****
18>42	729	729	172009	3.67E-12	3.67E-11	****
25>30	729	729	162378	1.25E-07	1.25E-06	****
25>37	729	729	122808	0.964	1	ns
25>42	729	729	133324	0.48	1	ns
30>37	729	729	65377	1	1	ns
30>42	729	729	92722	1	1	ns
37>42	729	729	122043	0.973	1	ns
**Asp-155-TAA**	**n1**	**n2**	**statistic**	**p-value**	**p-value.adj**	**p.adj.signif**
18>25	1273	1273	649876	8.99E-78	8.99E-77	****
18>30	1273	1273	596193	3.42E-48	3.42E-47	****
18>37	1273	1273	433201	0.017	0.172	ns
18>42	1273	1273	670695	3.4E-91	3.4E-90	****
25>30	1273	1273	359172	1	1	ns
25>37	1273	1273	259891	1	1	ns
25>42	1273	1273	625587	1.72E-63	1.72E-62	****
30>37	1273	1273	339173	1	1	ns
30>42	1273	1273	651642	7.21E-79	7.21E-78	****
37>42	1273	1273	658220	5.07E-83	5.07E-82	****
18>25	1273	1273	649876	8.99E-78	8.99E-77	****

Supplementary Table 3. Wilcoxon one-sided testing, adjusted using the Bonferroni method, shows statistical evidence of a non-linear temperature-driven effect on SCR: 18°C>25°C~37°C>42°C. We assessed the median of fluorescence relative to the wild-type for all the reporters studied in LB and M9 media (dataset from Fig S2), excluding those with no SCR at any of the studied temperatures. Source data is provided as Source data file.

T (°C)	n1	n2	statistic	p-value	p-value.adj	p.adj.signif
18>25	102	102	3731	4.5E-11	2.7E-10	****
18>37	102	102	4002	1.69E-12	1.01E-11	****
18>42	102	102	4098	1.18E-14	7.08E-14	****
25>37	102	102	800.5	0.227	1	ns
25>42	102	102	938	3.84E-05	0.00023	***
37>42	102	102	577	6.46E-05	0.000388	***

Supplementary Table 4. Mass spectrometry analysis of the reporters revealed that stop codon readthrough primarily related to amino acid misincorporations. TGA was almost always replaced by tryptophan (W) and, in lower frequency, by cysteine (C), glutamic acid (E), aspartic acid (D), and methionine (M). TAG was mainly replaced by glutamine (Q) and tyrosine (Y). Misincorporation of alanine (A), serine (S), tryptophan (W) and lysine (K) was a minor process. TAA was mainly replaced by glutamine (Q), tyrosine (Y) and alanine (A). Misincorporation of serine (S) and lysine (K) at the TAA position was a minor process. Major incorporations (relative abundance >10%) are shown in bold.

aa position mutated to stop codon	stop codon	peptides detected by ms[1]	misincorporated amino acids detected by ms	relative abundance (%)[2]
105	TAA	(R)VMNFEDGG**X**VTVTQDTSLEDGTLIYK(V)<br>(R)VMNFEDGG**K**	Q K **Y** A S	**Q(58), Y(15), A(26)**<br>S(<1), K
105	TAG	(R)VMNFEDGG**X**VTVTQDTSLEDGTLIYK(V)<br>(R)VMNFEDGG**K**	Q K Y A S W	**Q(77), Y(14),** A(9)<br>S(<1), W(<1), K
105	TGA	(R)VMNFEDGG**X**VTVTQDTSLEDGTLIYK(V)	W C	**W(99)**, C(1)
135	TAG	(K)LRGTNFPPDG**X**VMQK(K)<br>(R)GTNFPPDG**X**VMQK(K)<br>(K)LRGTNFPPDG**K**(V)<br>(R)GTNFPPDG**K**(V)	Q Y K	**Q(73), Y(27)**<br>K
135	TGA	(K)LRGTNFPPDG**X**VMQK(K)<br>(R)GTNFPPDG**X**VMQK(K)	W C	**W(99)**, C(1)
145	TAG	(K)TMGW**X**ASTER(L)<br>(K)KTMGW**X**ASTER(L)	Q Y W	**Q(39), Y(58),** W(2)
145	TGA	(K)TMG**X**ASTER(L)<br>(K)KTMGW**X**ASTER(L)	W E	**W(>99)**<br>E(<1)
155	TGA	(R)LYPE**X**GVLK(G)<br>(R)LYPE**X**GVLKGDIK(M)	W D	**W(>99)**<br>D(<1)
190	TGA	(K)KPVQ**X**PGAYNVDR(K)<br>(K)AKKPVQ**X**PGAYNVDR(K)	W C M	**W(99)**, C(~1)<br>M(<1)

[1] - Only peptides covering mutated position are shown; **X** or **K** (for Lys) designate misincorporated amino acid

[2] - Calculated as described in Materials and Methods for the corresponding forms of the peptide comprising mutated position; K was not included.

Supplementary Table 5. Mass spectrometric analysis of *E.coli* proteome identified peptides from non-coding regions resulted from stop codon readthrough events. Peptides indicating SCR events are listed with modification and charge state as reported by DIA-NN. TGA was more error-prone stop codon than TAA. We did not detect any evidences of SCR in TAG proteins, probable due to is low representation in the *E.coli* genome (8%). We detected more cases of SCR in *E.coli* samples grown at l8°C than at 37°C (14 vs. 11, see also Fig 2A and C).

Identified peptide[1]	Gene	Peptide type	Stop Codon	Canonical Protein Length(aa)	Peptide Start	Peptide End	Inserted aa	IDs 18°C	IDs 37°C	Found at 18°C[2]	Found at 37°C[2]	Median log2 Int 18°C[3]	Median log2 Int 37°C[3]	Log Change	P-value[4]	Significant[5]
(R)GSS**W**WSSVPLSDQMSR(R)	*astC*	Covers_Stop	TGA	406	403	418	W	6	0	TRUE	FALSE	19.5398			1	FALSE
(R)GVFAPLQIICVV(*)	*birA*	Past_Stop	TAA	321	324	335	X	4	6	TRUE	TRUE	16.9271	18.3338	-1.4067	0.0537	FALSE
(K)GPAAVNVTAI**W**SNPLI(*)	*cspC*	Covers_Stop	TGA	69	59	74	W	5	0	TRUE	FALSE	18.3445			1	FALSE
(R)NIAATLAIGMRNAG**M**QGR(A)	*garK*	Covers_Stop	TGA	381	367	384	M	3	6	TRUE	TRUE	15.5465	17.0081	-1.4616	0.0006	TRUE
(K)VLLIP**W**NR(G)	*gatD*	Covers_Stop	TGA	346	341	348	W	6	5	TRUE	TRUE	22.0872	19.6054	2.4818	0	TRUE
(K)SEDAMSTQLDPTQLAIEFLR(R)	*menI*	Past_Stop	TGA	136	166	185	X	2	3	FALSE	TRUE	17.3435	16.1639	1.1796	0.6136	FALSE
(R)KAGEAA**V**TVK(N)	*mhpF*	Covers_Stop	TGA	316	310	319	V	5	3	TRUE	TRUE	16.2749	17.9175	-1.6426	0.0079	FALSE
(K)AS**W**KPLLNLLP(*)	*ribB*	Covers_Stop	TGA	217	215	225	W	6	6	TRUE	TRUE	19.8146	18.1035	1.7111	0	TRUE
(K)VYAGNEHNHAAQQPQVLDI**C**SGL(*)	*rplM*	Covers_Stop	TAA	142	123	145	C	4	5	TRUE	TRUE	18.6309	16.5107	2.1202	0.0102	FALSE
(K)QPALGYLN**C**TPK(R)	*rpsG*	Covers_Stop	TGA	179	171	182	C	6	6	TRUE	TRUE	18.9541	18.8967	0.0574	0.6483	FALSE
(K)QPALGYLN**W**TPK(R)	*rpsG*	Covers_Stop	TGA	179	171	182	W	6	6	TRUE	TRUE	44.146	31.6105	12.5355	0.0119	FALSE
(R)DDEAEK**T**EINGVAK(C)	*ybaM*	Covers_Stop	TGA	53	47	60	T	1	4	FALSE	TRUE	20.9835	20.7976	0.1859	1	FALSE
(K)KVA**P**GQNIASSR(R)	*ybaQ*	Covers_Stop	TAA	113	110	121	P	3	2	TRUE	FALSE	20.6712	19.0116	1.6596	0.958	FALSE
(K)EITMGKTQPLPILITGGGR(R)	*ydgI*	Past_Stop	TAA	460	470	488	X	3	1	TRUE	FALSE	20.6283	18.9162	1.7121	1	FALSE
(K)LAHQAMTLK(L)	*yeiI*	Past_Stop	TAA	362	391	399	X	4	0	TRUE	FALSE	16.791			1	FALSE
(K)ILIS**F**IR(K)	*yrhD*	Covers_Stop	TAA	51	47	53	F	4	5	TRUE	TRUE	22.0012	21.6268	0.3744	0.189	FALSE

[1]Amino acids inserted at the Stop codon are **bold**, underlined residues are modified: carbamidomethylation (+57) at C, oxidation (+18) at M; preceding and following amino acids indicated in brackets, * indicates end of sequence. [2]Identification in a given condition (18 or 37ºC) is accepted if detected in at least 3 out of 6 replicates. [3]Median of log2 intensity values as reported by DIA-NN over replicates of the condition; if empty, peptide was not detected in the condition. [4]Unadjusted p-value of two-sided Student's t-test on log2-transformed intensities of the two temperature conditions. [5]After adjustment by Benjamini-Hochberg method

Supplementary Table 6. Summary of the mutants designed to experimentally test the effect of the nucleotides up- and downstream of the stop codon on SCR events. We mutated the nucleotide downstream of the stop codon to T and 5-nt upstream of the stop codon to ATTAT.

Premature stop codon position	WT sequence	Mutating the 1-nt dwonstream of the stop codon	Mutating the 5-nt upstream of the stop codon	Mutating the 1-nt dwonstream and the 1-nt downstream of the stop codon
105	GCGGC**TGA**G	GCGGC**TGAT**	**ATTATTGA**G	**ATTATTGAT**
135	ATGGC**TGA**G	ATGGC**TGAT**	**ATTATTGA**G	**ATTATTGAT**
155	CGGAA**TGA**C	CGGAA**TGAT**	**ATTATTGA**C	**ATTATTGAT**

Supplementary Table 7. Percentage of His-tag expression relative to the wild-type mScarlet and percentage of fluorescence signal measured as the median of fluorescence relative to the wild-type mScarlet.

Reporter name	% His-tag	% Fluorescence
Ala-105-taa	26.1138	0.0303
Ala-105-tag	16.6564	13.8490
Ala-105-tga	53.4456	81.2308
Ala-165-taa	0.0000	0.0769
Ala-165-tag	0.0000	0.3108
Ala-165-tga	13.8180	4.0235
Arg-150-taa	0.0000	0.0000
Arg-150-tag	0.0000	0.3755
Arg-150-tga	51.3889	4.1801
Asn-195-taa	0.0000	0.3064
Asn-195-tag	0.0000	0.0956
Asn-195-tga	0.0000	1.0808
Asp-155-taa	0.0000	0.1104
Asp-155-tag	0.0000	0.4369
Asp-155-tga	9.1593	18.0366
Asp-170-taa	0.0000	0.0000
Asp-170-tag	0.0000	0.0042
Asp-170-tga	0.0000	0.0094
Gln-110-taa	0.0000	0.2310
Gln-110-tag	0.0000	1.6252
Gln-110-tga	43.2341	0.0235
Glu-11-taa	22.3912	0.0024
Glu-11-tag	0.0000	0.0032
Glu-11-tga	0.0000	0.0650
Glu-115-taa	0.0000	0.0072
Glu-115-tga	0.0000	0.6391
Glu-145-taa	0.0000	0.0224
Glu-145-tag	0.0000	0.0610
Glu-145-tga	3.1135	5.5713
Glu-31-taa	0.0000	0.0134
Glu-31-tag	0.0000	0.0822
Glu-31-tga	7.1517	0.6052
Glu-90-taa	0.0000	0.3587
Glu-90-tag	0.0000	0.0568
Glu-90-tga	19.2487	3.0498
Glu-95-taa	0.0000	0.0024
Glu-95-tag	0.0000	0.0000
Glu-95-tga	0.0000	2.5026
Gly-160-taa	0.0000	0.0000
Gly-160-tag	0.0000	0.0000
Gly-160-tga	33.9755	0.0000
Gly-21-taa	0.0000	0.0000
Gly-21-tag	0.0000	0.0000
Gly-21-tga	0.0000	0.0000
Gly-36-taa	0.0000	0.0000
Gly-36-tag	0.0000	0.0000
Gly-36-tga	7.1570	0.0078
His-205-taa	0.0000	0.0099
His-205-tag	0.0000	0.0287
His-205-tga	0.0000	1.6275
His-26-taa	0.0000	0.0070
His-26-tag	0.0000	0.0635
His-26-tga	0.0000	1.6590
Ile-120-tag	0.0000	0.0056
Ile-120-tga	0.0000	0.0022
Ile-61-tag	0.0000	0.0029
Ile-80-taa	0.0000	0.0037
Ile-80-tag	0.0000	0.0148
Ile-80-tga	1.4096	0.9731
Leu-125-taa	0.0000	0.0047
Leu-125-tag	0.0000	0.0020
Leu-125-tga	0.0000	0.5865
Leu-175-taa	0.0000	0.0085
Leu-175-tag	0.0000	0.0004
Leu-175-tga	1.3221	0.6199
Leu-200-taa	0.0000	0.0000
Leu-200-tag	0.0000	0.0000
Leu-200-tga	2.4623	0.0000
Lys-140-taa	0.0000	0.0032
Lys-140-tag	0.0000	0.0593
Lys-140-tga	11.2558	2.0226
Lys-16-taa	0.0000	0.0883
Lys-16-tag	0.0000	0.2042
Lys-16-tga	0.0000	0.3331
Lys-185-taa	0.0000	0.0000
Lys-185-tag	0.0000	0.0050
Lys-185-tga	0.0000	0.0030
Lys-46-tag	0.0000	0.0320
Lys-46-tga	0.0000	0.7955
Lys-75-taa	0.0000	0.0077
Lys-75-tag	0.0000	0.0067
Lys-75-tga	11.2602	5.9544
Lys-85-taa	0.0000	0.0022
Lys-85-tag	0.0000	0.0198
Lys-85-tga	0.0000	0.0006
Met-13-taa	0.0000	0.0249
Met-13-tag	0.0000	0.0057
Met-13-tga	9.4340	0.0037
Met-19-taa	0.0000	0.0434
Met-19-tag	0.0000	0.0233
Met-19-tga	0.0000	0.0024
Met-190-taa	0.0000	0.0110
Met-190-tag	48.6070	0.2116
Met-190-tga	74.7775	0.2116
Phe-100-taa	0.0000	0.0044
Phe-100-tag	0.0000	0.0079
Phe-100-tga	0.0000	0.0037
Phe-130-taa	0.0000	0.0000
Phe-130-tag	0.0000	0.0056
Phe-130-tga	0.0000	5.2781
Pro-135-taa	0.0000	0.2827
Pro-135-tag	2.6279	5.1460
Pro-135-tga	82.5845	78.6557
Pro-56-taa	0.0000	0.0174
Pro-56-tag	0.0000	0.0154
Pro-56-tga	0.0000	2.7410
Thr-180-taa	0.0000	0.0006
Thr-180-tag	0.0000	0.006
Thr-180-tga	0.0000	0.0008
Thr-210-taa	0.0000	0.1463
Thr-210-tag	0.0000	0.2253
Thr-210-tga	0.0000	0.2590
Trp-94-taa	0.0000	0.0019
Trp-94-tag	0.0000	0.0000
Trp-94-tga	73.3500	57.5179

Supplementary Table 8. **OD values of mScarlet wild-type *E. coli* cultures grown in a 384-well plate without shaking under various temperature and media conditions**, offering insights into growth dynamics in response to environmental factors. The data represent three biological replicates.

Temperature (°C)	media	Mean OD	SD OD
18	LB	0.67	0.21
25	LB	2.37	0.30
30	LB	2.16	0.51
37	LB	3.20	0.25
42	LB	1.99	0.71
18	M9	0.10	0.03
25	M9	0.35	0.32
30	M9	0.30	0.10
37	M9	0.56	0.04
42	M9	0.29	0.02

Supplementary References

1. Searle, B. C. *et al.* Generating high quality libraries for DIA MS with empirically corrected peptide predictions. *Nat. Commun.* **11**, 1548 (2020).
2. Tierrafría, V. H. *et al.* RegulonDB 11.0: Comprehensive high-throughput datasets on transcriptional regulation in Escherichia coli K-12. *Microb Genom* **8**, (2022).
3. Gessulat, S. *et al.* Prosit: proteome-wide prediction of peptide tandem mass spectra by deep learning. *Nat. Methods* **16**, 509–518 (2019).
