# Supplementary material for: Environment modulates protein heterogeneity through transcriptional and translational stop codon readthrough
Source: Nat Commun. 2024 May 24;15:4446. doi: 10.1038/s41467-024-48387-x (PMC11126739; doi:10.1038/s41467-024-48387-x)
Supplement: Supplementary file 3 — Description of Additional Supplementary Files [file 41467_2024_48387_MOESM3_ESM.pdf]

Description of Additional Supplementary Files

Supplementary Data 1. Sequencing data of the RNA-seq experiments (RNAseq.zip).

- Ala105taa_bwa_sorted_only_mapped.bam
- Ala105tag_bwa_sorted_only_mapped.bam
- Ala105tga_bwa_sorted_only_mapped.bam
- Asp155tga_bwa_sorted_only_mapped.bam
- Glu145tag_bwa_sorted_only_mapped.bam
- Glu145tga_bwa_sorted_only_mapped.bam
- Met190tga_bwa_sorted_only_mapped.bam
- Pro056taa_bwa_sorted_only_mapped.bam
- Pro056tag_bwa_sorted_only_mapped.bam
- Pro056tga_bwa_sorted_only_mapped.bam
- Pro135tag_bwa_sorted_only_mapped.bam
- Pro135tga_bwa_sorted_only_mapped.bam
- Reference_to_plasmidASK_mScarlet.fa
- WT_bwa_sorted_only_mapped.bam

Supplementary Data 2. Sequencing data of the DNA-seq experiments (DNA-seq.zip).

- Pro-56-taa-F-Premixed
- Pro-56-tag-F-Premixed
- Pro-56-tga-F-Premixed
- Ala-105-taa-F-Premixed.ab1
- Ala-105-tag-F-Premixed.ab1
- Ala-105-tga-F-Premixed.ab1
- Pro-135-tag-F-Premixed.ab1
- Pro-135-tga-F-Premixed.ab1
- Glu-145-tag-F-Premixed.ab1
- Glu-145-tga-F-Premixed.ab1
- Asp-155-tga-F-Premixed.ab1
- Met-190-tga-F-Premixed.ab1

Supplementary Data 3. **Number of cells imaged and used to derive the fluorescence distributions of Fig. 2C**
