# Supplementary material for: Environment modulates protein heterogeneity through transcriptional and translational stop codon readthrough
Source: Nat Commun. 2024 May 24;15:4446. doi: 10.1038/s41467-024-48387-x (PMC11126739; doi:10.1038/s41467-024-48387-x)
Supplement: Supplementary file 7 — Reporting Summary [file 41467_2024_48387_MOESM7_ESM.pdf]

nature portfolio

Corresponding author(s): Maria Luisa Romero Romero
Agnes Toth-Petroczy

Last updated by author(s): 28/03/2024

Reporting Summary

Nature Portfolio wishes to improve the reproducibility of the work that we publish. This form provides structure for consistency and transparency in reporting. For further information on Nature Portfolio policies, see our Editorial Policies and the Editorial Policy Checklist.

Statistics

For all statistical analyses, confirm that the following items are present in the figure legend, table legend, main text, or Methods section.

n/a	Confirmed	
☐	☒	The exact sample size (*n*) for each experimental group/condition, given as a discrete number and unit of measurement
☐	☒	A statement on whether measurements were taken from distinct samples or whether the same sample was measured repeatedly
☐	☒	The statistical test(s) used AND whether they are one- or two-sided<br>*Only common tests should be described solely by name; describe more complex techniques in the Methods section.*
☐	☒	A description of all covariates tested
☐	☒	A description of any assumptions or corrections, such as tests of normality and adjustment for multiple comparisons
☐	☒	A full description of the statistical parameters including central tendency (e.g. means) or other basic estimates (e.g. regression coefficient) AND variation (e.g. standard deviation) or associated estimates of uncertainty (e.g. confidence intervals)
☐	☒	For null hypothesis testing, the test statistic (e.g. *F*, *t*, *r*) with confidence intervals, effect sizes, degrees of freedom and *P* value noted<br>*Give P values as exact values whenever suitable.*
☒	☐	For Bayesian analysis, information on the choice of priors and Markov chain Monte Carlo settings
☒	☐	For hierarchical and complex designs, identification of the appropriate level for tests and full reporting of outcomes
☒	☐	Estimates of effect sizes (e.g. Cohen's *d*, Pearson's *r*), indicating how they were calculated

Our web collection on statistics for biologists contains articles on many of the points above.

Software and code

Policy information about availability of computer code

Data collection	E. coli protein sequences extracted from UniProt database (version October 2022)
Data analysis	Image analysis: Fiji ImageJ v.2.0.0-rc-69/1.5p,R v4.1.2<br>mScarlet mass spectometry analysis: MASCOT v.2.2.04, Scaffold (v.4.11.1, Proteome Software, Portland), Xcalibur software (Thermo Fischer Scientific),<br>RNA-seq: BWA v0.7.17-r1198, Samtools mpileup v1.15.1 using htslib 1.15.1, PySam v0.16.0 with Python 3.7.12, R v4.1.2.<br>Analysis of E. coli proteome by mass spectrometry: DIA-NN software suit v1.8, Proteowizard MSConvert v3.0.2, Skyline 'daily', version 22.2.1.351, Python 3<br>RNA secondary structures prediction: RNAfold2

For manuscripts utilizing custom algorithms or software that are central to the research but not yet described in published literature, software must be made available to editors and reviewers. We strongly encourage code deposition in a community repository (e.g. GitHub). See the Nature Portfolio guidelines for submitting code & software for further information.

Data

Policy information about availability of data

All manuscripts must include a data availability statement. This statement should provide the following information, where applicable:

- Accession codes, unique identifiers, or web links for publicly available datasets
- A description of any restrictions on data availability
- For clinical datasets or third party data, please ensure that the statement adheres to our policy

The mass spectrometry data have been deposited in the MassIVE repository under accession number MSV000091065 (Login: MSV000091065_reviewer and password: k64sa21). The raw fluorescent data obtained from microscopy images and the Western blot gels are provided as Source Data files. RNA-seq data were deposited to GEO (accession number: GSE226936) and are provided as Supplementary Data 2 (BAM files) and their DNA-seq chromatograms as Supplementary Data 3 (bl1 files).

Research involving human participants, their data, or biological material

Policy information about studies with human participants or human data. See also policy information about sex, gender (identity/presentation), and sexual orientation and race, ethnicity and racism.

Reporting on sex and gender	NA
Reporting on race, ethnicity, or other socially relevant groupings	NA
Population characteristics	NA
Recruitment	NA
Ethics oversight	NA

Note that full information on the approval of the study protocol must also be provided in the manuscript.

Field-specific reporting

Please select the one below that is the best fit for your research. If you are not sure, read the appropriate sections before making your selection.

☒ Life sciences ☐ Behavioural & social sciences ☐ Ecological, evolutionary & environmental sciences

For a reference copy of the document with all sections, see nature.com/documents/nr-reporting-summary-flat.pdf

Life sciences study design

All studies must disclose on these points even when the disclosure is negative.

Sample size	We acquired bright-field and fluorescence images of E. coli samples. The sample size corresponds to the number of segmented cells using the bright-field images (listed in Supplementary Table 9)
Data exclusions	Images with scratches or with less than ten cells segmented were excluded
Replication	All attemps of replication lead to the same results (2 or 3 biological replicates, Supplementary Table 9)
Randomization	The allocation of samples into 384-well plates was random
Blinding	During data collection and analyses researches were blinding to group allocation

Reporting for specific materials, systems and methods

We require information from authors about some types of materials, experimental systems and methods used in many studies. Here, indicate whether each material, system or method listed is relevant to your study. If you are not sure if a list item applies to your research, read the appropriate section before selecting a response.

Materials & experimental systems

n/a	Involved in the study	
☐	☒	Antibodies
☒	☐	Eukaryotic cell lines
☒	☐	Palaeontology and archaeology
☐	☒	Animals and other organisms
☒	☐	Clinical data
☒	☐	Dual use research of concern
☒	☐	Plants

Methods

n/a	Involved in the study	
☒	☐	ChIP-seq
☒	☐	Flow cytometry
☒	☐	MRI-based neuroimaging

Antibodies

Antibodies used	Qiagen mouse anti-penta His-tag (# 34660) and Licor IRDYE 800 goat anti mouse (#926-32350)
Validation	https://www.qiagen.com/us/products/discovery-and-translational-research/protein-purification/tagged-protein-expression-purification-detection/anti-his-antibodies-bsa-free?catno=34660

Animals and other research organisms

Policy information about studies involving animals; ARRIVE guidelines recommended for reporting animal research, and Sex and Gender in Research

Laboratory animals	wild-type K-12 MG1655 E. coli strain
Wild animals	NA
Reporting on sex	NA
Field-collected samples	NA
Ethics oversight	NA

Note that full information on the approval of the study protocol must also be provided in the manuscript.

Plants

Seed stocks	NA
Novel plant genotypes	NA
Authentication	NA
