# Supplementary material for: Environment modulates protein heterogeneity through transcriptional and translational stop codon readthrough
Source: Nat Commun. 2024 May 24;15:4446. doi: 10.1038/s41467-024-48387-x (PMC11126739; doi:10.1038/s41467-024-48387-x)
Supplement: Supplementary file 8 — Source Data [file 41467_2024_48387_MOESM8_ESM.zip › Source_Data_file/Source_Data_files_explanation.docx]

- Source Data file Fig1C corresponding to Figure 1C.
- Source Data file Fig1E – fluorescence measurements of cells grown at 37°C in LB corresponding to Figure 1E.
- Source Data file Fig2AB_EF – % of median fluorescence relative to wild-type of cells grown at 18°C, 25°C, 37°C and 42°C in LB and M9 corresponding to Figure 2A, B, E and F.
- Source Data file Fig2C – fluorescence measurements of cells transformed with reporters introducing stop codons at positions 105 and 155 grown at 18°C, 25°C, 37°C and 42°C in LB corresponding to Figure 2C.
- Source Data file Fig3A_3B – nucleotide frequency in a 5-nt window upstream and downstream of the stop codon vs the SCM score corresponding to Figure 3AB.
- Source Data file Fig3C – nucleotide frequency in a 1-nt window upstream and downstream of the stop codon vs low, mid and high SCM score corresponding to Figure 3C.
- Source Data file Fig3E – His-tag expression in cells transformed with reporters introducing TGA at position 105, 135 and 150, as well as their mutants, corresponding to Figure 3E.
- Source Data files Fig3Egel1and Fig3Egel2 – Western blot gels reveling the His-tag expression in cells transformed with reporters introducing TGA at position 105, 135 and 150, as well as their mutants, corresponding to Figure 3E.
- Source Data file Fig4AB – distributions of mRNA mismatch percentages for nucleotides encoding amino acids, premature stop codons, and canonical stop codons, corresponding to Figure 4AB.
- Source Data file Fig4CD –boxplots of mRNA mismatch percentages for nucleotides encoding amino acids, premature stop codons, and canonical stop codons depending of the adjacent nucleotides, corresponding to Figure 4CD.
- Source Data file Fig4E – amino acid encoded by the mRNA sequence at the premature stop codon versus the number of reads, corresponding to Figure 4E.
- Source Data file Fig5A – amino acid misinserted by SCM events at the TAA and TGA stop codon of the *E. coli* proteome, corresponding to Figure 5A.
- Source Data file Fig5B – occurrence of an additional stop codon within specific window sizes downstream of the stop codon in the *E. coli* genome of the K12 MG1655 strain, corresponding to Figure 5B.
- Source Data file Fig5D – nucleotide identity of the position following the stop codon that experiences SCM in the *E. coli* proteome, corresponding to Figure 5D.
- Source Data file FigS1 – fluorescence measurements within the linear dynamic range of the microscope, corresponding to Figure S1.
- Source Data file FigS2A – fluorescence measurements of cells grown at 18°C in LB corresponding to Figure S2A.
- Source Data file FigS2B – fluorescence measurements of cells grown at 25°C in LB corresponding to Figure S2B.
- Source Data file FigS2C – fluorescence measurements of cells grown at 37°C in LB corresponding to Figure S2C.
- Source Data file FigS2D – fluorescence measurements of cells grown at 42°C in LB corresponding to Figure S2D.
- Source Data file FigS2E – fluorescence measurements of cells grown at 18°C in M9 corresponding to Figure S2E.
- Source Data file FigS2F – fluorescence measurements of cells grown at 25°C in M9 corresponding to Figure S2F.
- Source Data file FigS2G – fluorescence measurements of cells grown at 37°C in M9 corresponding to Figure S2G.
- Source Data file FigS2H – fluorescence measurements of cells grown at 42°C in M9 corresponding to Figure S2H.
- Source Data file FigS3A – percentage of reporters dispaying SCM events, corresponding to Figure S3A.
- Source Data file FigS3B – box plots summarising fluorescence distributions of *E. coli* cells expressing nine selected reporters grown under various conditions, corresponding to Figure S3B.
- Source Data file FigS3C – fluorescence measurements of the mScarlet thermostability assay, corresponding to Figure S3C.
- Source Data file FigS4 – subdataset of the dataset shown in Fig 2C analyzed by Wilcoxon test, corresponding to Figure S4.
- Source Data file FigS5 – subdataset of the dataset shown in Fig 2B andF analyzed by Wilcoxon test, corresponding to Figure S5.
- Source Data file FigS6A – fluorescence measurements of cells grown at 37°C in M9 supplemented with higher carbon sourse, corresponding to Figure S6A.
- Source Data file FigS6B – fluorescence measurements of cells grown at 37°C in M9 supplemented with higher casaamino acids, corresponding to Figure S6B.
- Source Data file FigS8
- Source Data file FigS9
- Source Data file FigS10 – SCM score versus amino acids position, corresponding to Figure S10.
- Source Data file FigS11A – versus SCM score versus predicted minimum free energy (MFE) in a 100-nt window upstreams of the stop codon, corresponding to Figure S11A.
- Source Data file FigS11B – versus SCM score versus predicted minimum free energy (MFE) in a 100-nt window downstream of the stop codon, corresponding to Figure S11A.
- Source Data file FigS11C – predicted minimum free energy (MFE) versus low, mid and high SCM score in a 100-nt window upstreams and downstream of the stop codon, corresponding to Figure S11C.
- Source Data file FigS11D – SCM score versus number of pairs in in various size windows upstreams and of the stop codon, corresponding to Figure S11D.
- Source Data file FigS11E – SCM score versus number of pairs in in various size windows downstream and of the stop codon, corresponding to Figure S11E.
- Source Data file FigS13_S17 – RNA polymerase mismatches versus amino acid position, corresponding to Figure S13 and S17.
- Source Data file FigS15 – occurrence of an additional stop codon within specific window sizes downstream of the stop codon in the *E. coli* genome of the BL21 strain, corresponding to Figure 5B.
- Source Data file FigS16A – percentage of stop codon identity at following categories: 1) *Single gene operons*, 2) *Final multi-gene operons*, and 3) *Within multi-gene operons*, corresponding to Figure S16A.
- Source Data file FigS16A – percentage of stop codon identity in the *E. coli* genome at following categories: 1) *Single gene operons*, 2) *Final multi-gene operons*, and 3) *Within multi-gene operons*, corresponding to Figure S16A.
- Source Data file FigS16B – percentage of the nucleotide identity after TGA in the *E. coli* genome at following categories: 1) *Single gene operons*, 2) *Final multi-gene operons*, and 3) *Within multi-gene operons*, corresponding to Figure S16A.
- Source Data file FigS18
