# Supplementary figures and images for: Environment modulates protein heterogeneity through transcriptional and translational stop codon readthrough
Source: Nat Commun. 2024 May 24;15:4446. doi: 10.1038/s41467-024-48387-x (PMC11126739; doi:10.1038/s41467-024-48387-x)

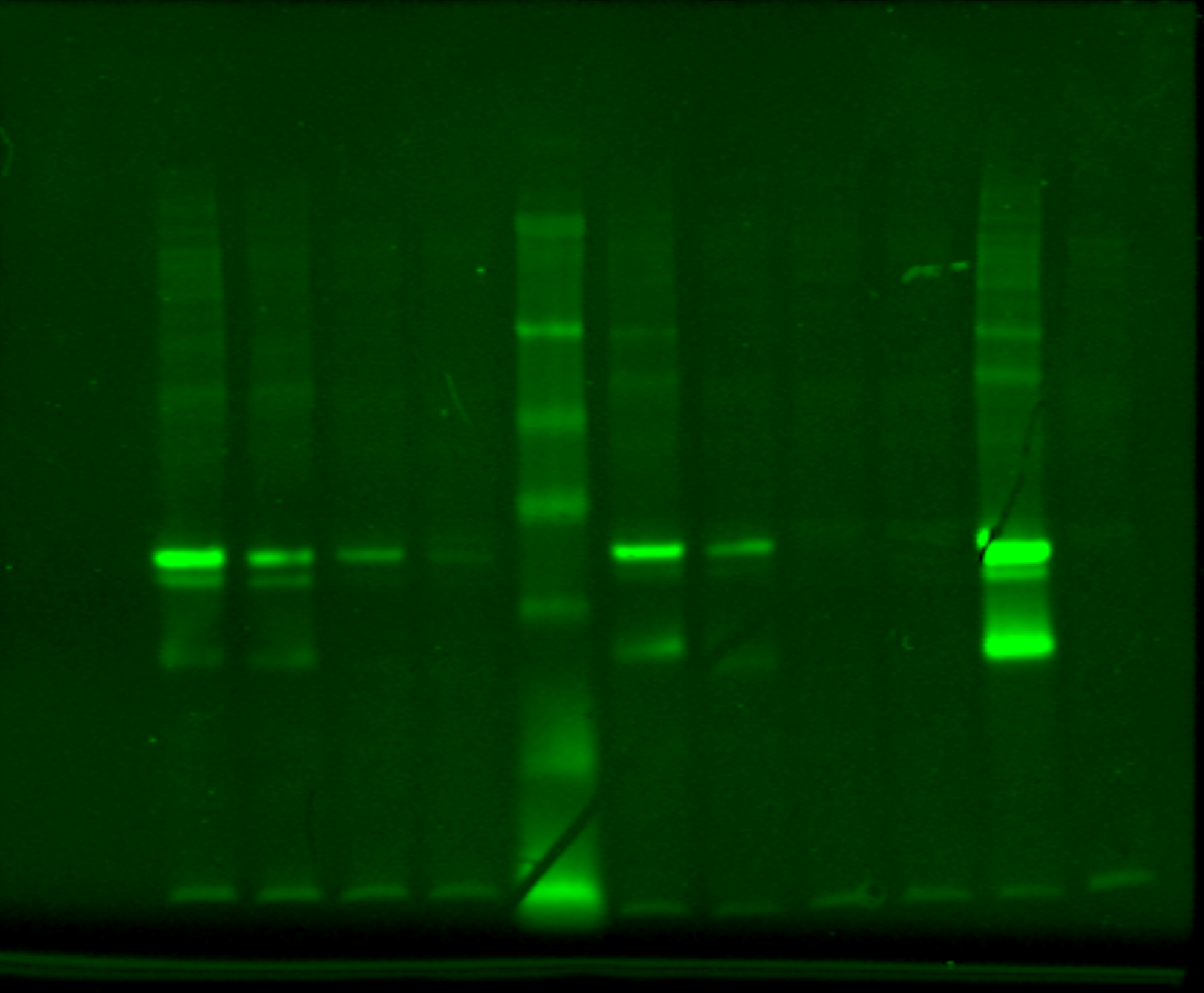

Supplement: Supplementary file 8 — Source Data [file 41467_2024_48387_MOESM8_ESM.zip › Source_Data_file/Fig3/Fig3Egel1.tif]

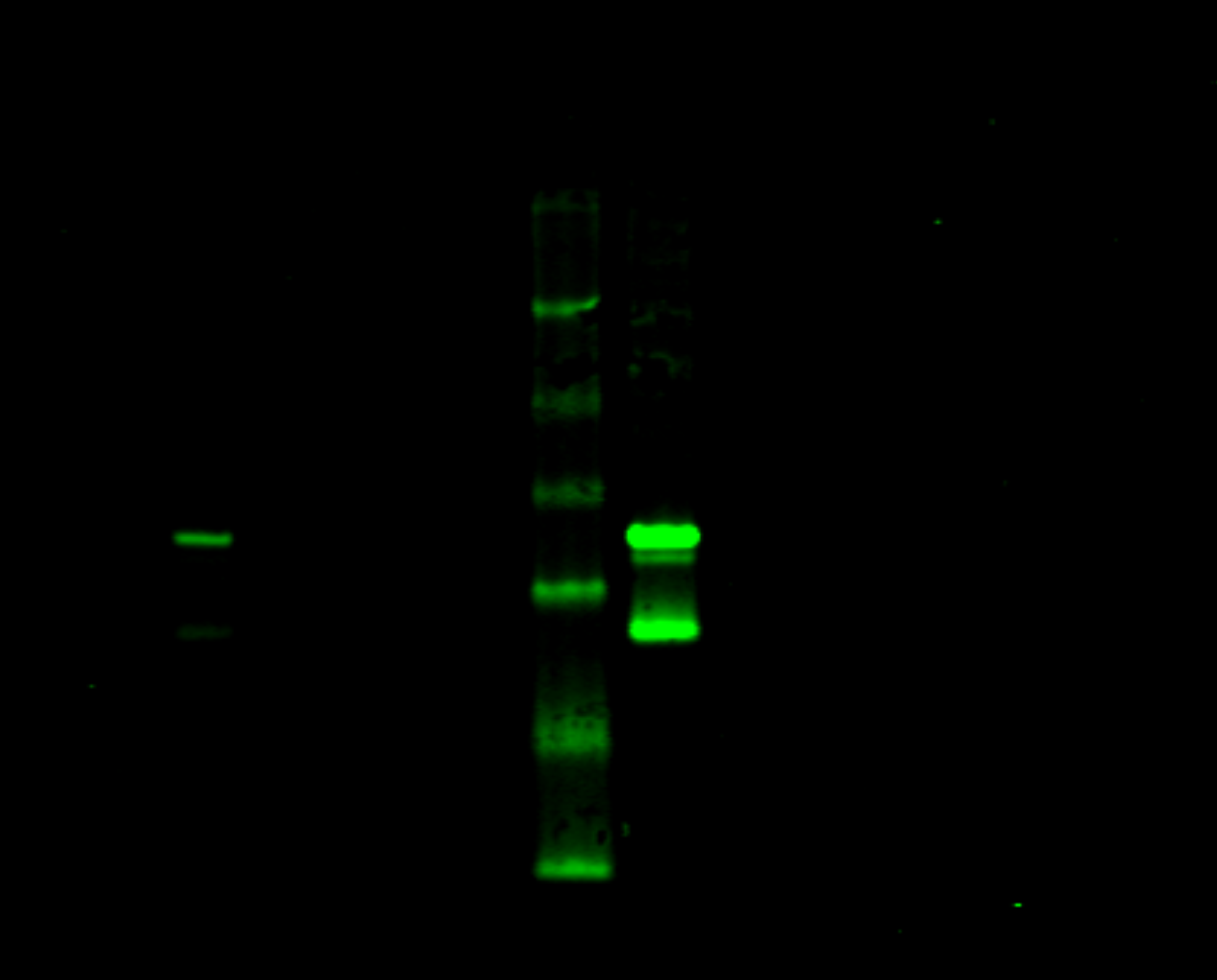

Supplement: Supplementary file 8 — Source Data [file 41467_2024_48387_MOESM8_ESM.zip › Source_Data_file/Fig3/Fig3Egel2.tif]

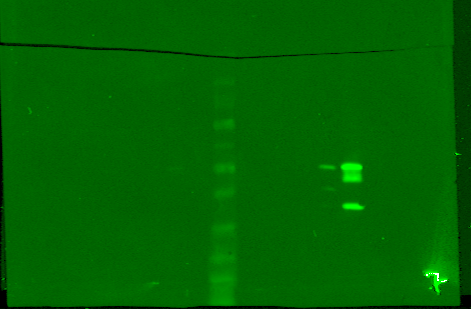

Supplement: Supplementary file 8 — Source Data [file 41467_2024_48387_MOESM8_ESM.zip › Source_Data_file/FigS8/FigS8Gel10_800.tif]

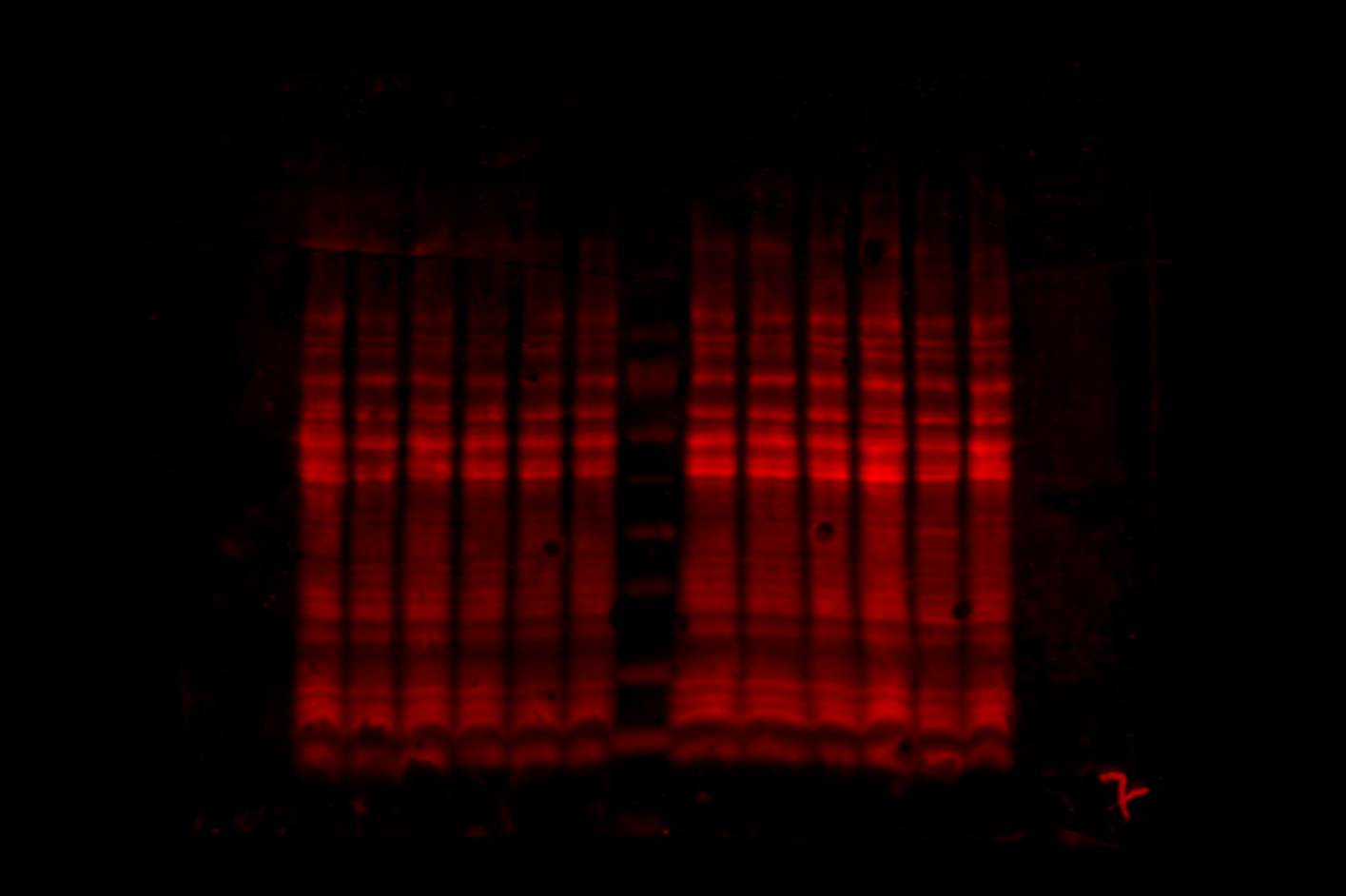

Supplement: Supplementary file 8 — Source Data [file 41467_2024_48387_MOESM8_ESM.zip › Source_Data_file/FigS8/FigS8Gel10_total_proteins.tif]

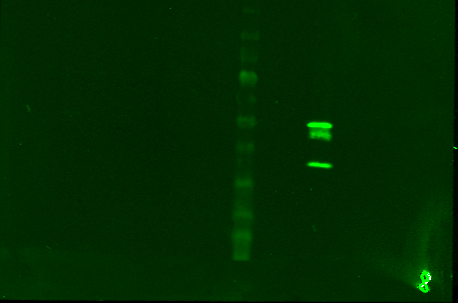

Supplement: Supplementary file 8 — Source Data [file 41467_2024_48387_MOESM8_ESM.zip › Source_Data_file/FigS8/FigS8Gel11_800.tif]

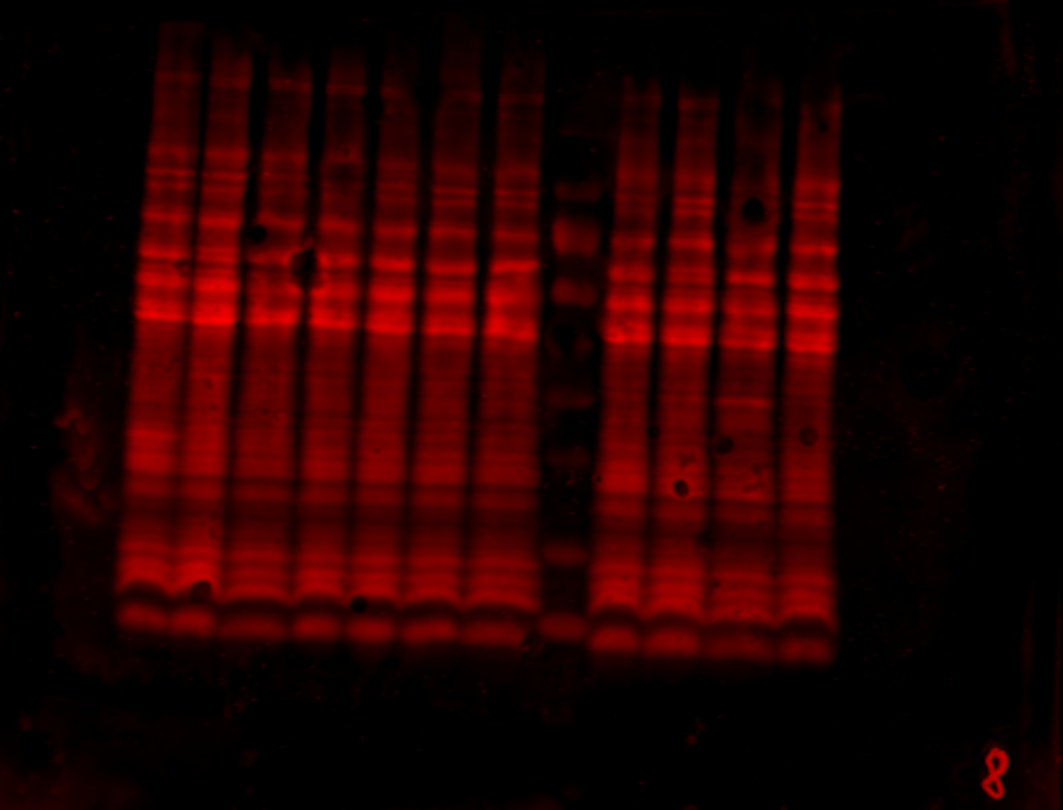

Supplement: Supplementary file 8 — Source Data [file 41467_2024_48387_MOESM8_ESM.zip › Source_Data_file/FigS8/FigS8Gel11_total_proteins.tif]

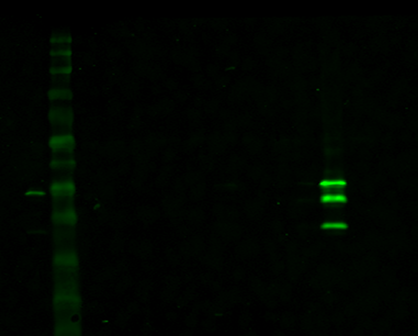

Supplement: Supplementary file 8 — Source Data [file 41467_2024_48387_MOESM8_ESM.zip › Source_Data_file/FigS8/FigS8Gel12_800.tif]

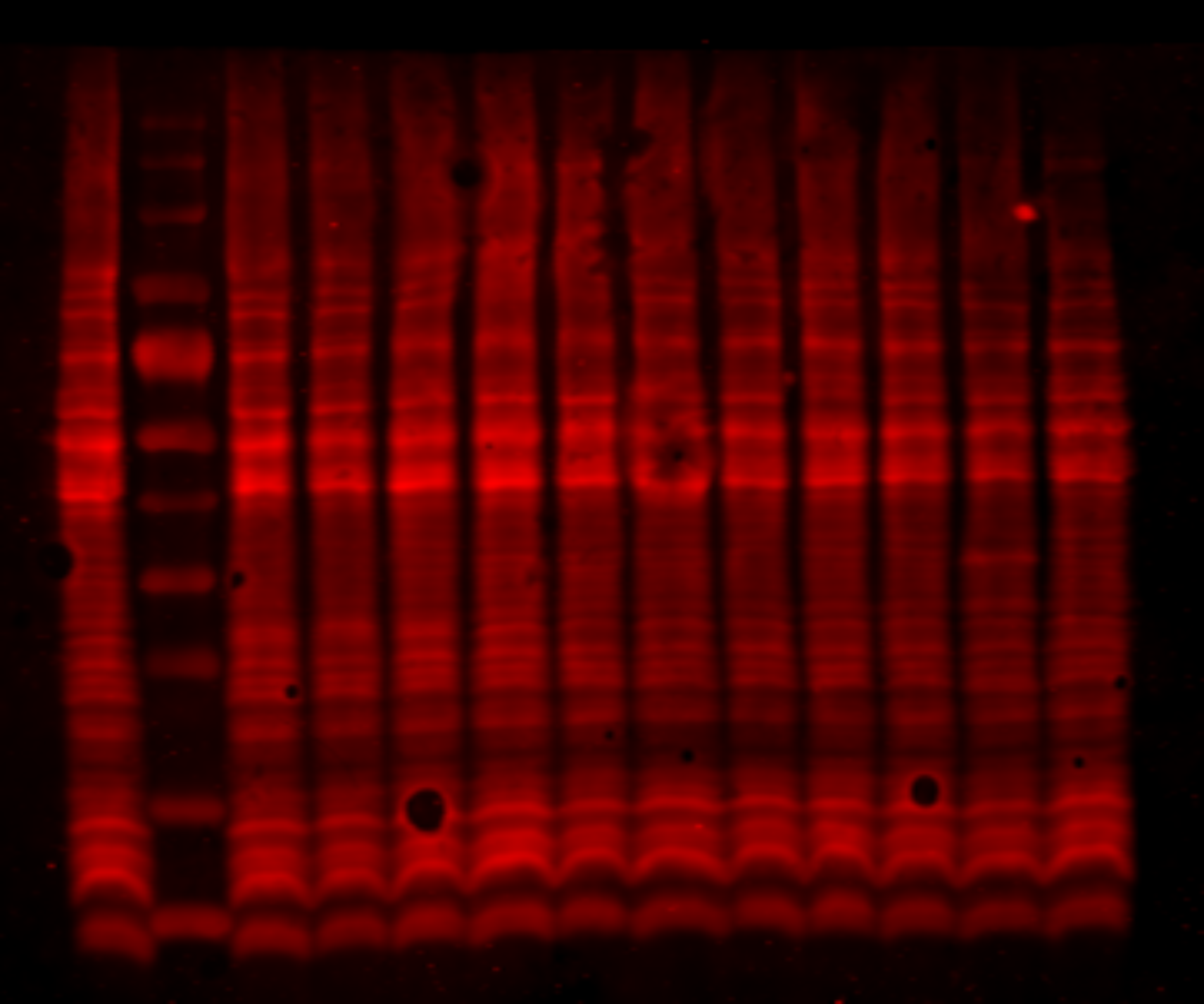

Supplement: Supplementary file 8 — Source Data [file 41467_2024_48387_MOESM8_ESM.zip › Source_Data_file/FigS8/FigS8Gel12_total_proteins.tif]

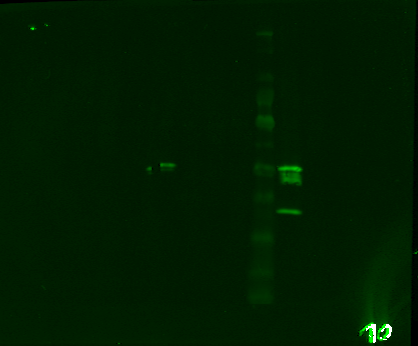

Supplement: Supplementary file 8 — Source Data [file 41467_2024_48387_MOESM8_ESM.zip › Source_Data_file/FigS8/FigS8Gel13_800.tif]

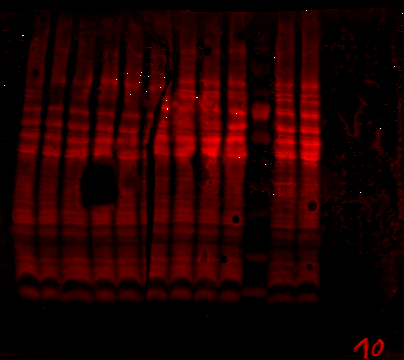

Supplement: Supplementary file 8 — Source Data [file 41467_2024_48387_MOESM8_ESM.zip › Source_Data_file/FigS8/FigS8Gel13_total_proteins.tif]

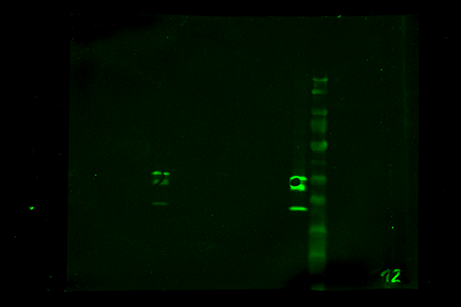

Supplement: Supplementary file 8 — Source Data [file 41467_2024_48387_MOESM8_ESM.zip › Source_Data_file/FigS8/FigS8Gel14_800.tif]

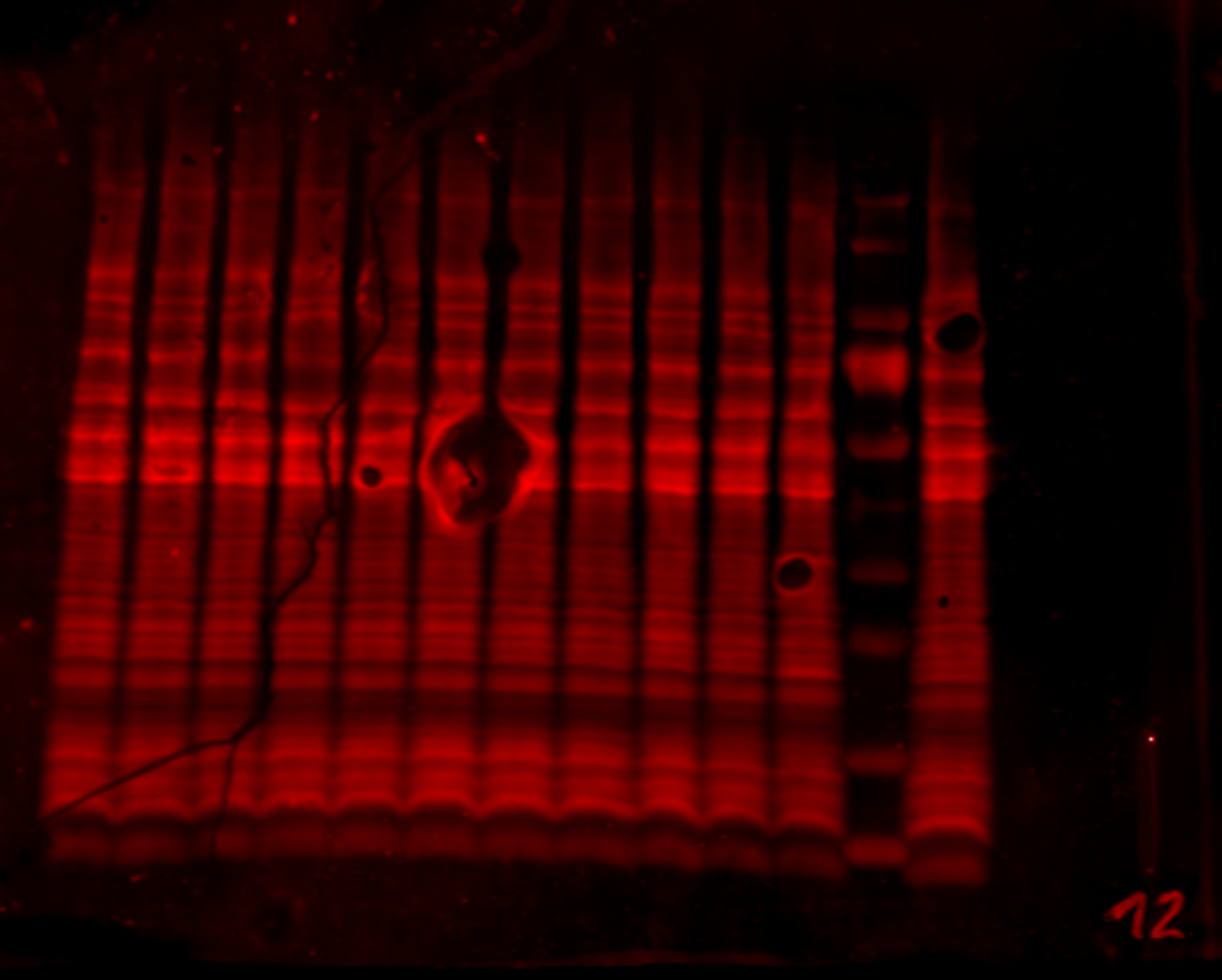

Supplement: Supplementary file 8 — Source Data [file 41467_2024_48387_MOESM8_ESM.zip › Source_Data_file/FigS8/FigS8Gel14_total_proteins.tif]

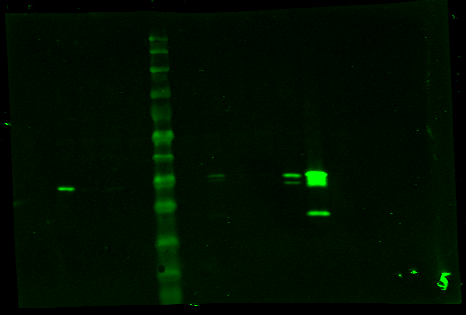

Supplement: Supplementary file 8 — Source Data [file 41467_2024_48387_MOESM8_ESM.zip › Source_Data_file/FigS8/FigS8Gel1_800.tif]

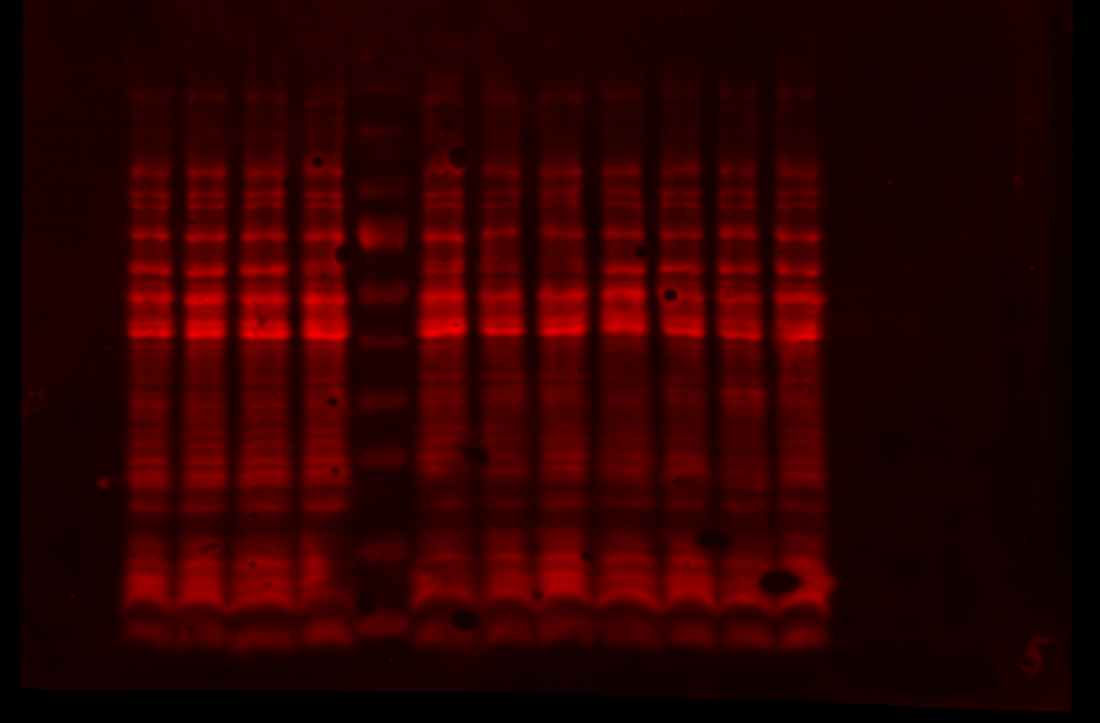

Supplement: Supplementary file 8 — Source Data [file 41467_2024_48387_MOESM8_ESM.zip › Source_Data_file/FigS8/FigS8Gel1_total_proteins.tif]

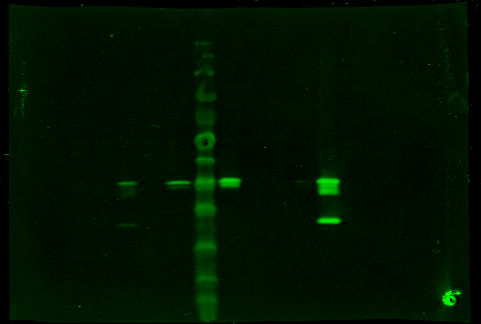

Supplement: Supplementary file 8 — Source Data [file 41467_2024_48387_MOESM8_ESM.zip › Source_Data_file/FigS8/FigS8Gel2_800.tif]

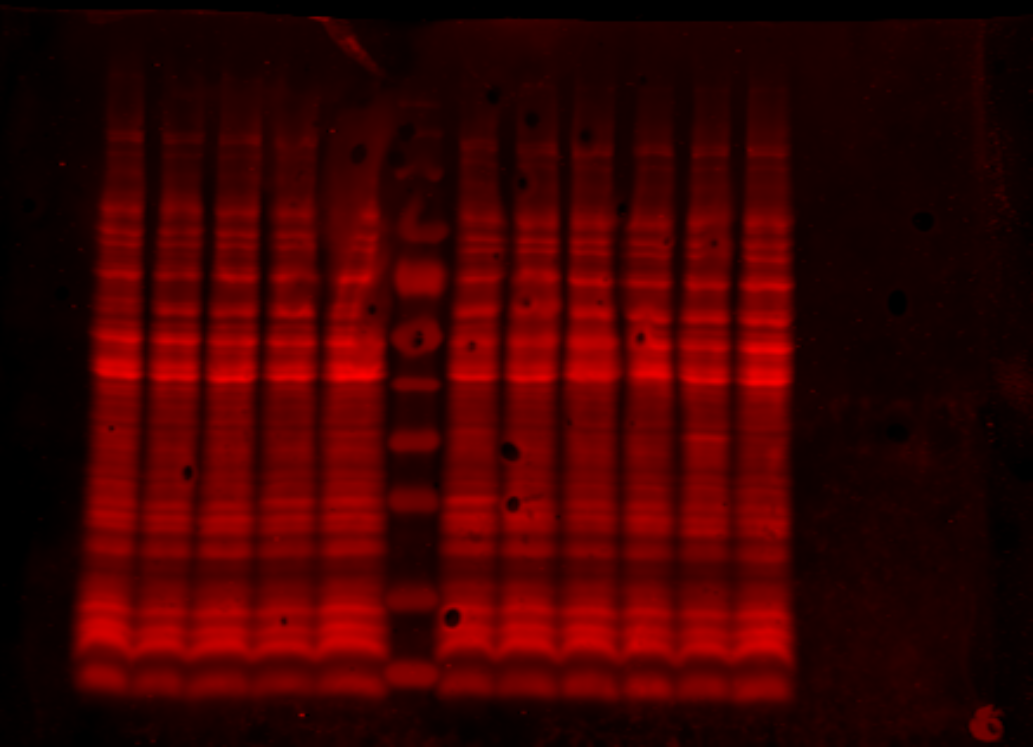

Supplement: Supplementary file 8 — Source Data [file 41467_2024_48387_MOESM8_ESM.zip › Source_Data_file/FigS8/FigS8Gel2_total_proteins.tif]

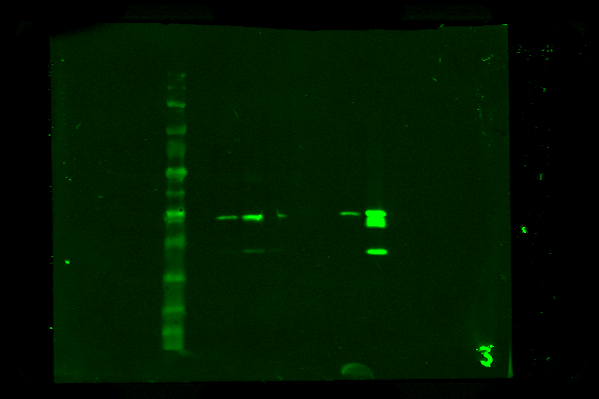

Supplement: Supplementary file 8 — Source Data [file 41467_2024_48387_MOESM8_ESM.zip › Source_Data_file/FigS8/FigS8Gel3_800.tif]

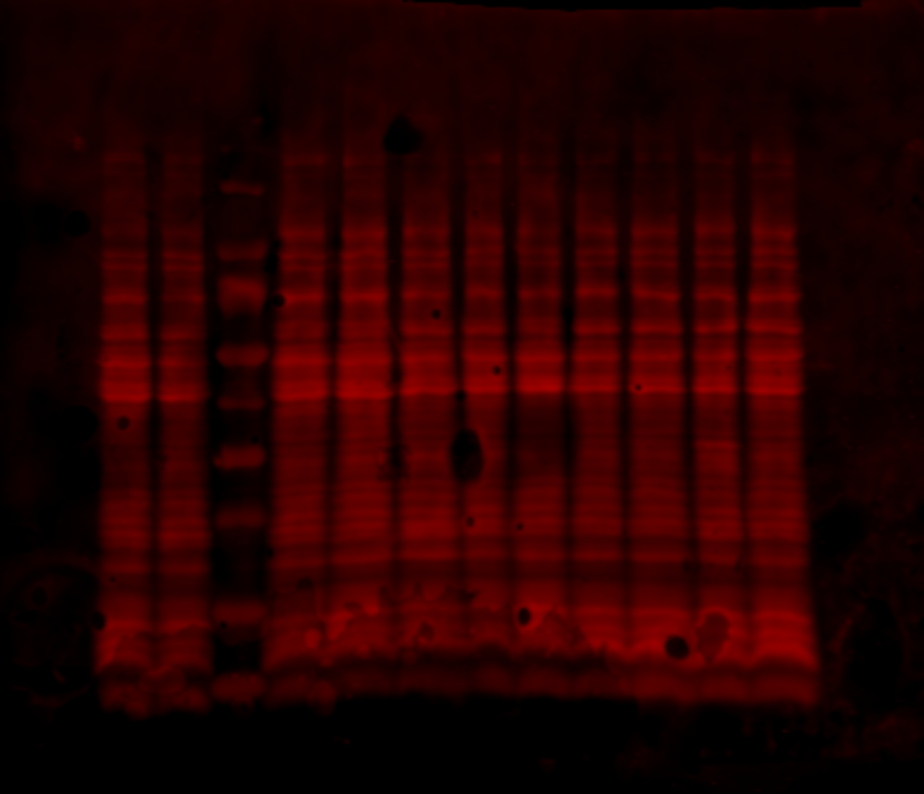

Supplement: Supplementary file 8 — Source Data [file 41467_2024_48387_MOESM8_ESM.zip › Source_Data_file/FigS8/FigS8Gel3_total_proteins.tif]

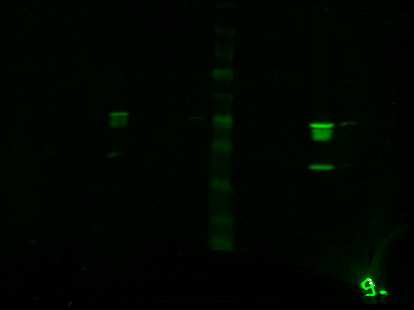

Supplement: Supplementary file 8 — Source Data [file 41467_2024_48387_MOESM8_ESM.zip › Source_Data_file/FigS8/FigS8Gel4_800.tif]

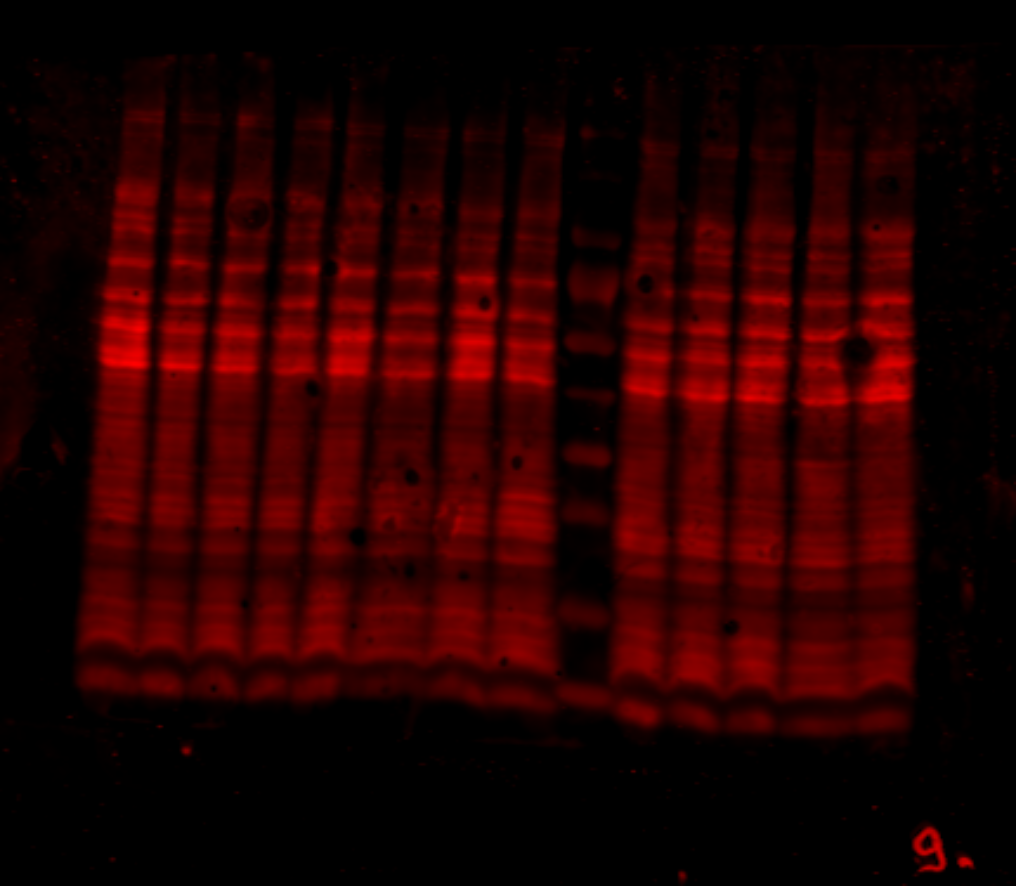

Supplement: Supplementary file 8 — Source Data [file 41467_2024_48387_MOESM8_ESM.zip › Source_Data_file/FigS8/FigS8Gel4_total_proteins.tif]

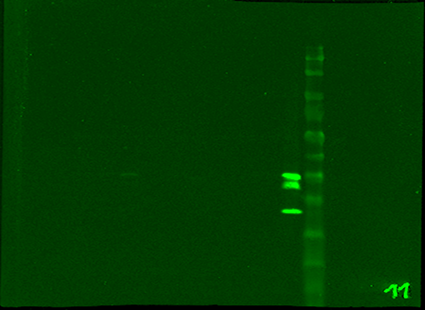

Supplement: Supplementary file 8 — Source Data [file 41467_2024_48387_MOESM8_ESM.zip › Source_Data_file/FigS8/FigS8Gel5_800.tif]

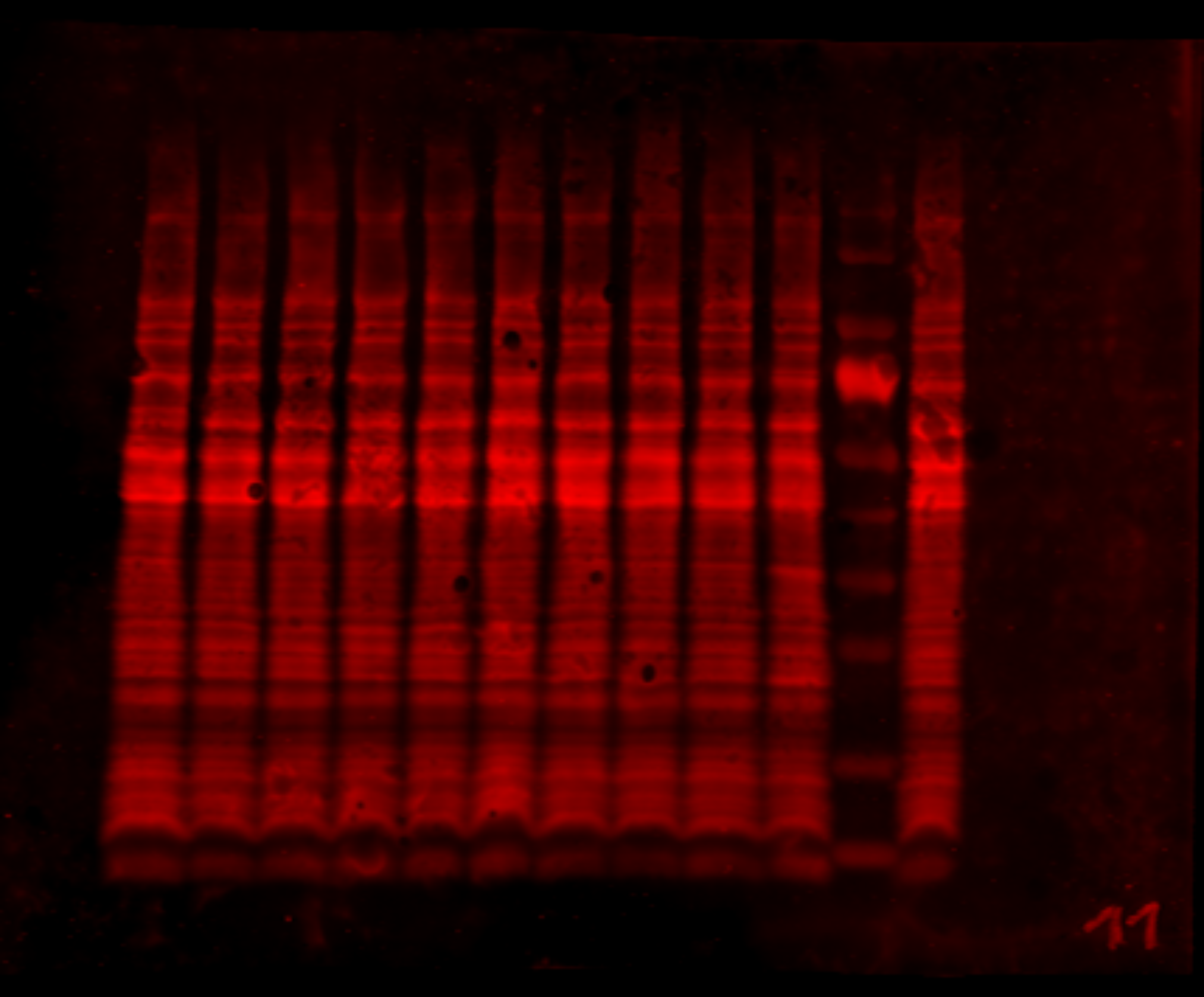

Supplement: Supplementary file 8 — Source Data [file 41467_2024_48387_MOESM8_ESM.zip › Source_Data_file/FigS8/FigS8Gel5_total_proteins.tif]

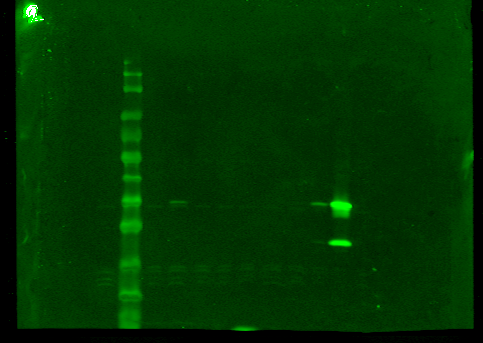

Supplement: Supplementary file 8 — Source Data [file 41467_2024_48387_MOESM8_ESM.zip › Source_Data_file/FigS8/FigS8Gel6_800.tif]

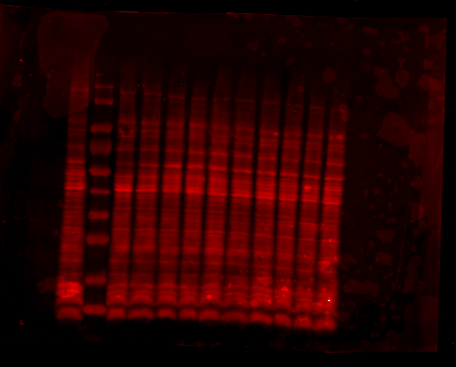

Supplement: Supplementary file 8 — Source Data [file 41467_2024_48387_MOESM8_ESM.zip › Source_Data_file/FigS8/FigS8Gel6_total_proteins.tif]

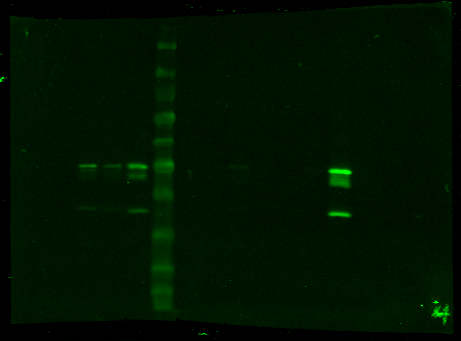

Supplement: Supplementary file 8 — Source Data [file 41467_2024_48387_MOESM8_ESM.zip › Source_Data_file/FigS8/FigS8Gel7_800.tif]

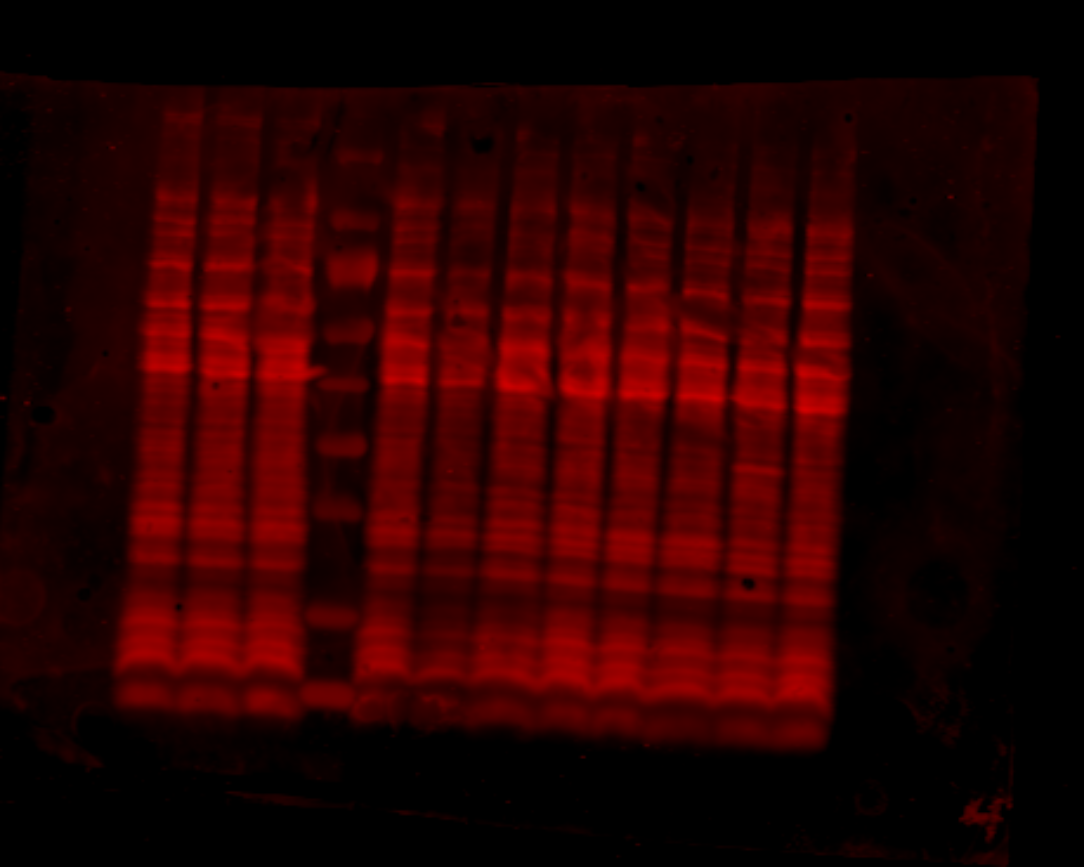

Supplement: Supplementary file 8 — Source Data [file 41467_2024_48387_MOESM8_ESM.zip › Source_Data_file/FigS8/FigS8Gel7_total_proteins.tif]

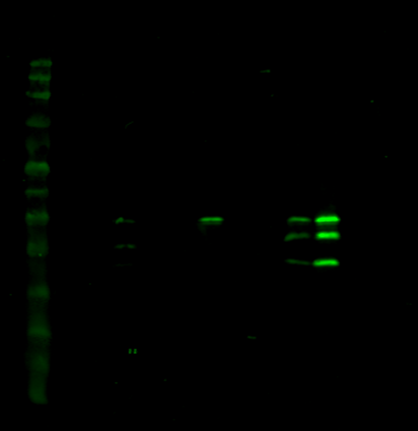

Supplement: Supplementary file 8 — Source Data [file 41467_2024_48387_MOESM8_ESM.zip › Source_Data_file/FigS8/FigS8Gel8_800.tif]

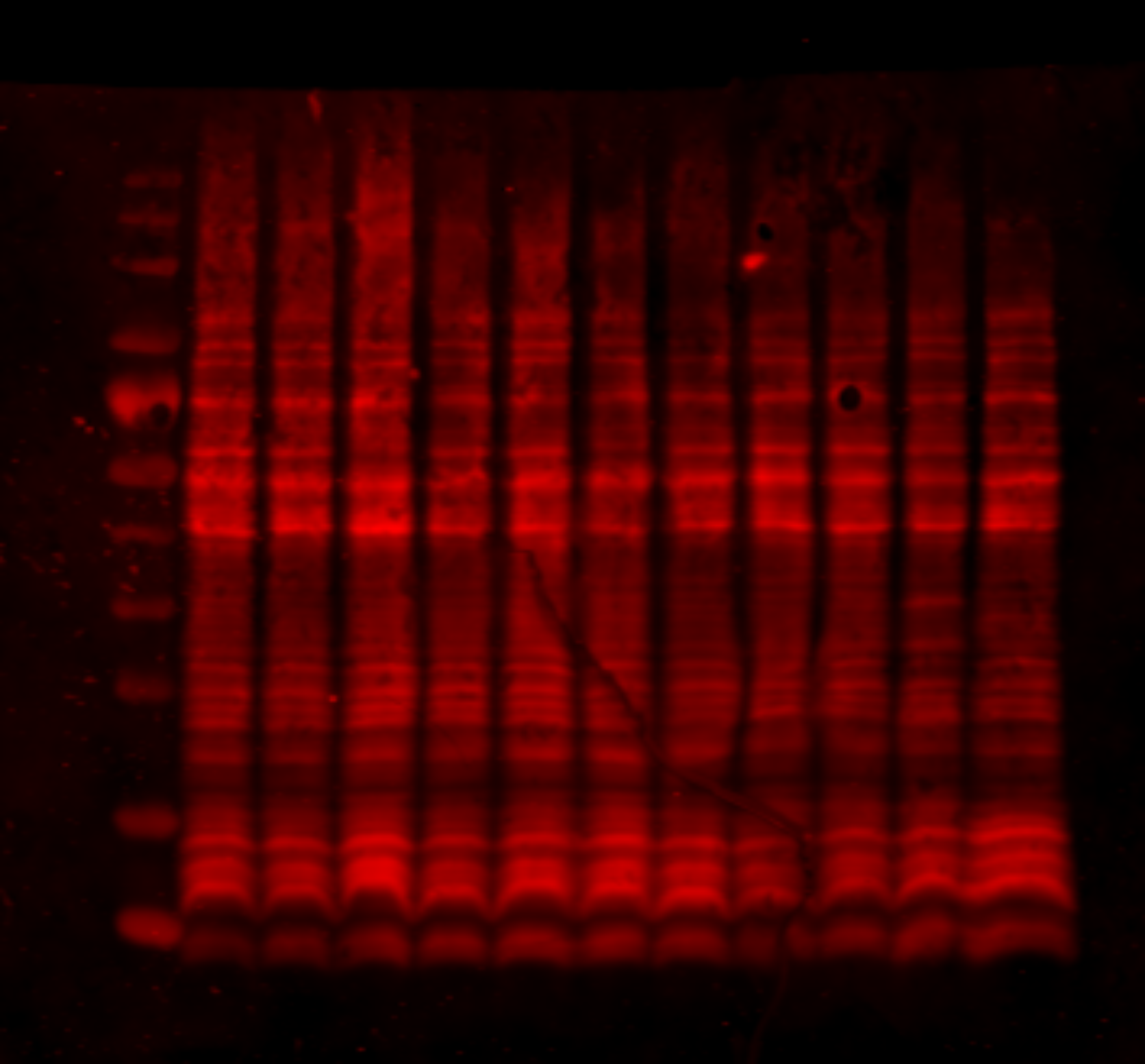

Supplement: Supplementary file 8 — Source Data [file 41467_2024_48387_MOESM8_ESM.zip › Source_Data_file/FigS8/FigS8Gel8_total_proteins.tif]

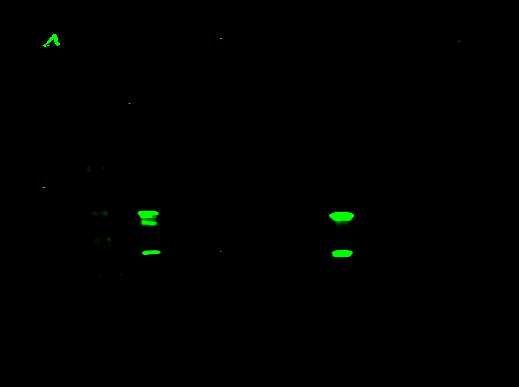

Supplement: Supplementary file 8 — Source Data [file 41467_2024_48387_MOESM8_ESM.zip › Source_Data_file/FigS8/FigS8Gel9_800.tif]

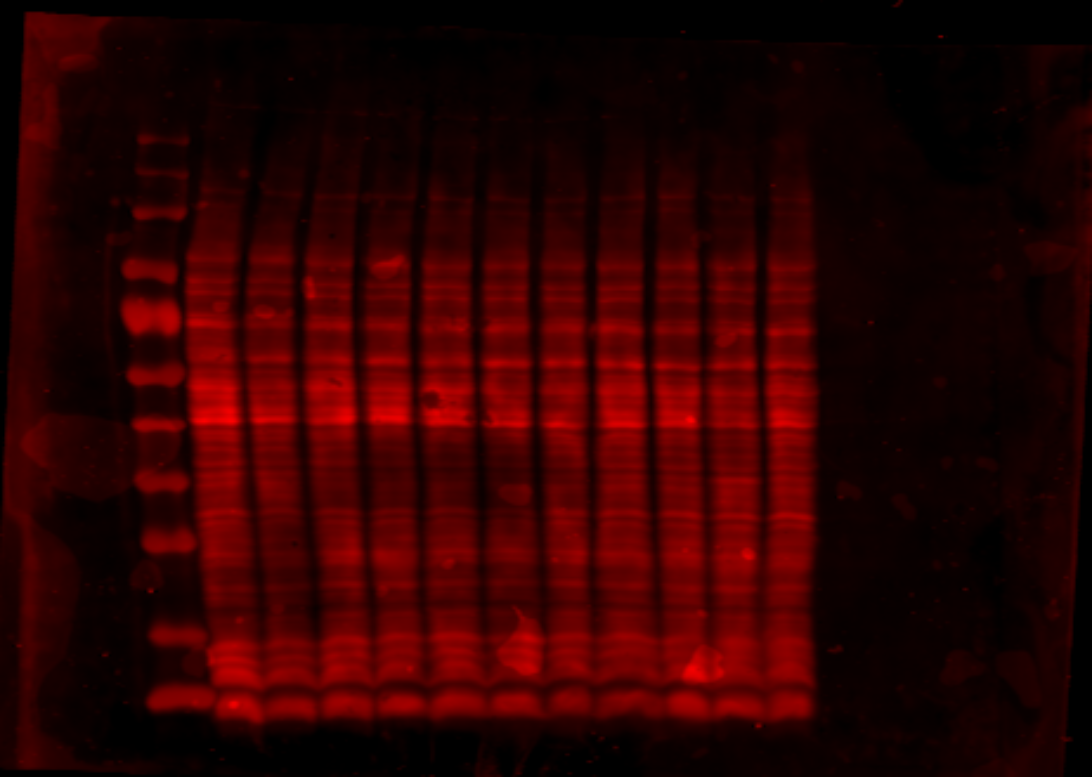

Supplement: Supplementary file 8 — Source Data [file 41467_2024_48387_MOESM8_ESM.zip › Source_Data_file/FigS8/FigS8Gel9_total_proteins.tif]
